# Unit-to-unit transfer due to shortage of intensive care beds in Sweden 2015–2019 was associated with a lower risk of death but a longer intensive care stay compared to no transfer: a registry study

**DOI:** 10.1186/s40560-024-00722-6

**Published:** 2024-02-27

**Authors:** Christian Rylander, Jesper Sternley, Max Petzold, Jonatan Oras

**Affiliations:** 1 Anaesthesiology and Intensive Care Medicine, Department of Surgical Sciences, Uppsala University and Uppsala University Hospital, 715 85 Uppsala, Sweden; 2 School of Public Health and Community Medicine, Institute of Medicine, University of Gothenburg, Gothenburg, Sweden; 3 Department of Anaesthesiology and Intensive Care Medicine, Clinical Sciences, University of Gothenburg and Sahlgrenska University Hospital, Gothenburg, Sweden

**Keywords:** Intensive care unit, Patient transfer, Transportation of patients, Logistics, Mortality, ICU transfer, Inter-hospital transfer, Intensive care, Critical care, Transport

## Abstract

**Background:**

Intensive care unit-to-unit transfer due to temporary shortage of beds is increasing in Sweden. Transportation induces practical hazards, and the change of health care provider may prolong the length of stay in intensive care. We previously showed that the risk of death at 90 days did not differ between patients transferred due to a shortage of beds and non-transferred patients with a similar burden of illness in a tertiary intensive care unit. The aim of this study was to widen the analysis to a nation-wide cohort of critically ill patients transferred to another intensive care unit in Sweden due to shortage of intensive care beds.

**Methods:**

Retrospective comparison between capacity transferred and non-transferred patients, based on data from the Swedish Intensive Care Registry during a 5-year period before the COVID-19 pandemic. Patients with insufficient data entries or a recurring capacity transfer within 90 days were excluded. To assess the association between capacity transfer and death as well as intensive care stay within 90 days after ICU admission, logistic regression models with step-wise adjustment for SAPS3 score, primary ICD-10 ICU diagnosis and the number of days in the intensive care unit before transfer were applied.

**Results:**

From 161,140 eligible intensive care admissions, 2912 capacity transfers were compared to 135,641 discharges or deaths in the intensive care unit. Ninety days after ICU admission, 28% of transferred and 21% of non-transferred patients were deceased. In the fully adjusted model, capacity transfer was associated with a lower risk of death within 90 days than no transfer; OR (95% CI) 0.71 (0.65–0.69) and the number of days spent in intensive care was longer: 12.4 [95% CI 12.2–12.5] vs 3.3 [3.3–3.3].

**Conclusions:**

Intensive care unit-to-unit transfer due to shortage of bed capacity as compared to no transfer during a 5-year period preceding the COVID-19 pandemic in Sweden was associated with lower risk of death within 90 days but with longer stay in intensive care.

**Supplementary Information:**

The online version contains supplementary material available at 10.1186/s40560-024-00722-6.

## Background

The number of patients being transferred between Swedish intensive care units (ICUs) due to temporary shortage of available beds has been increasing over the last decade: from 0.83% of all admissions in 2009 to 1.73% in 2019 [1]. During the COVID-19 pandemic 2020–2021, there was an even higher need for redistribution of ICU patients in Sweden as in several other countries [2–4]. However, for several years before the pandemic, the relatively low number of ICU beds in Sweden has been a major factor driving the numbers of capacity transfers due to lack of available ICU beds [5, 6]. An additional incitement is the growing centralization of specialized procedures to tertiary centres, necessitating more frequent outbound transfers from such units [7].

Transportation of a critically ill patient induces numerous practical hazards, for better not associated with increased mortality as they are compensated for by the professionals in charge of the transport [8, 9]. However, discharge to another ICU due to bed shortage, capacity transfer, has been associated with a higher 30-day mortality compared to transfer categorized as clinical or repatriation [10]. Focusing on the first category with the highest mortality, we previously showed for patients submitted to outbound capacity transfers from tertiary intensive care that their risk of death within 90 days did not differ from non-transferred patients with a similar burden of disease [11]. However, tertiary centre patients only represent a minority of all secondary transfers induced by the bed shortage which temporarily arise in most ICUs. In the present study, we expanded our analysis to unit-to-unit capacity transfers within the entire nation under non-pandemic conditions.

## Patients and methods

The study was approved by the Swedish Ethical Review Authority on December 18, 2020 (Dnr 2020-07089). Patient consent was waived due to the nature of the retrospective analysis of existing data. The manuscript was prepared according to the Strengthening the Reporting of Observational Studies in Epidemiology (STROBE) guidelines [12].

### Referral and transport procedures

There are no official Swedish guidelines for patient unit-to-unit referral or transportation during ongoing intensive care, but the Swedish Society of Anaesthesia and Intensive Care issued standard of care recommendations in 2015. Swedish hospitals offering intensive care service are all organised within or in adherence to the public health care system, using similar standard operating procedures (SOPs). When there is a need for unit-to-unit referral due to bed shortage, a patient is selected in dialogue with the next admitting unit based on the physiological reserve for a safe transport and the capacity for adequate care in the new hospital. Once a patient is selected, the transport is prepared using local checklists to avoid hazards and handover reports are given by telephone. Most of the capacity transfers are inter-hospital transfers but intra-hospital transfers occur, mainly between ICUs located within a major hospital. Such transports are carried out by the ordinary ICU personnel. For inter-hospital transports, the prehospital road and air ambulance systems are used, operating on an advanced level of equipment and competency [13]. For ground transport, a nurse specialised in intensive care or anaesthesia is joining the prehospital team and if necessary, an ICU physician is added. Securely fastened medical devices (ventilator, automatic syringes) and monitoring equipment (pulse-oximeter, electrocardiogram, arterial line with continuous blood pressure) and infusion pumps are powered from the vehicle and not dependent on batteries. The air-lift services capable of transporting intensive care patients are all manned by specially trained physicians and nurses.

### Study population

A 5-year study period from January 1, 2015 to December 31, 2019 preceding the COVID-19 pandemic was chosen to reflect ordinary conditions of intensive care and unit-to-unit transfers in Sweden. Patients > 18 years admitted to intensive care as identified in the Swedish Intensive Care Registry were eligible for the study. Exposure was defined as a unit-to-unit capacity transfer with a confirmed admission in the next admitting ICU, registered within 12 h after the time of discharge. Comparison was made to patients with similar age and burden of disease, remaining and cared for in the referring ICU until discharged to a ward or deceased. Erroneous registrations, admissions ending with discharge to home after a very short ICU stay or unit-to-unit transfers for other than capacity reasons were excluded as well as new capacity transfers or ICU readmissions within 90 days after discharge to a ward.

### Registry data

The Swedish Intensive Care Registry (www.icuregswe.org) is an open quality registry with a full coverage of the 83 Swedish ICUs reporting average of 45.896 yearly admittances 2015–2019. Unit-to-unit patient transfer due to shortage of ICU beds (lack of resources) is one of eight basic national quality indicators compulsory to report. In the registry, transfers of intensive care patients are categorised as (1) “clinical” when there is need for specialised care not available in the admitting hospital, (2) “repatriation” when patients are returned or forwarded to the ICU closest to their home address, or (3) “capacity transfer” when it is due to a shortage of staffed beds in the referring ICU. Data retrieval was formally applied for and granted by the board of the registry. The following variables were obtained: age, sex, date and time of admission, SAPS3, surgical status (elective surgery, acute surgery, no surgery), primary ICU diagnosis (ICD-10 code), date and time of discharge, discharge destination (specified ward or ICU), transfer category. The time of death for diseased patients as derived from the Swedish Population Register was included in the data set. The complete list of ICD codes is presented in Additional file 1.

### Outcomes and analyses

The outcomes of interest were survival status at 90 days after admission to the referring ICU and the total number of days spent in intensive care within 90 days after ICU admission. In the primary analysis we compared all capacity transfers to no transfer. In a secondary analysis we compared the respective inter-hospital and intra-hospital capacity transfers to no transfer.

The comparison of survival status was adjusted for confounders in four steps: Unadjusted; Model (1) adjusted for SAPS 3; Model (2) adjusted for SAPS 3 and primary ICD-10 ICU diagnosis; and Model (3) adjusted for SAPS 3, primary ICD-10 ICU diagnosis and days in the ICU before the capacity transfer/no transfer.

The comparisons of days spent in the ICU within 90 days after admission were adjusted using these steps except for Model 3 due to an anticipated interdependence between the number of days spent in the ICU and the time of death when occurring in the ICU.

Two sensitivity analyses were performed, with inclusion of additional variables in Model 3: (1) being transferred on readmission and (2) standardised mortality rate (SMR) in transferring and receiving ICU. Standardised mortality rate is a measure of unit performance, defined by the actual mortality divided by the expected mortality according to the SAPS3.

One additional post-hoc analysis was performed, with comparison limited to non-transferred patients being present in the ICU at the time of a transfer due to lack of resources.

### Statistics

Continuous variables were checked for symmetry. Symmetrically distributed variables are presented as the mean (standard deviation; SD) and non-symmetrically distributed variables are presented as median (Q1;Q3). The two-sided Student’s *t* test was used for comparison of means of symmetrically distributed variables, and the Mann–Whitney *U* test was used for comparison of distributions of non-symmetrically distributed variables. Fisher´s exact test was used for comparison of binary variables with dichotomous outcomes. The risk of death, expressed as the event odds ratio (OR), and the total number of days in the ICU within 90 days were compared between groups by applying multivariable logistic regression and Poisson regression, respectively, to the models. All variables, as presented above, were included in each model in the regression analysis with the exposed/non-exposed variable. IBM SPSS version 24.0 was used for the analysis.

## Results

### Patient cohort

**Fig. 1:**
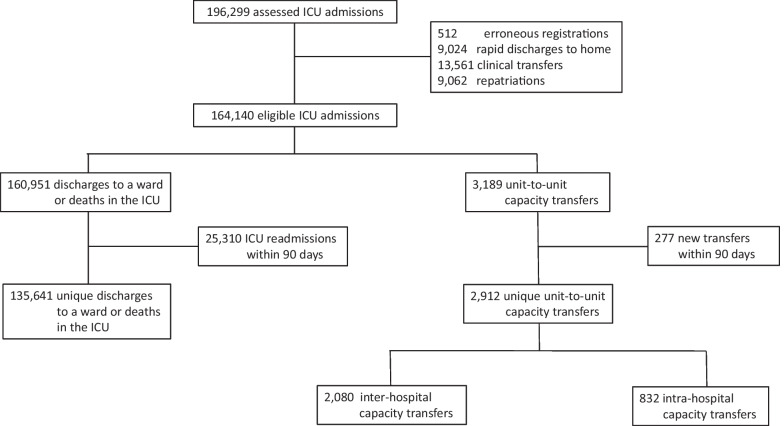
Study flow chart

Among 196,299 ICU admissions between January 1st 2015 and December 31, 2019, there were 32,159 meeting the primary exclusion criteria. From the remaining 3189 capacity transfers and 160,951 discharges to a ward or deaths in the ICU, another respective 277 and 25,310 were excluded due to a new transfer or to ICU readmission within 90 days. The final analysis included 2912 unique capacity transfers, of which 2080 were inter-hospital, and 135,641 unique discharges to a ward or deaths in the ICU (Fig. 1).

### Patient characteristics

The median (Q1;Q3) age of all 138,553 patients was 68 (54;76) years, and 56,995 (41%) were women. The most common ICU diagnosis was infection/sepsis (14,265; 10%), followed by respiratory failure including COPD/asthma (12.212; 8.8%) and intoxication (9272; 6.7%). Capacity transferred patients had higher SAPS 3, a higher estimated standardised mortality rate (SMR), presented had a higher burden of care and had spent shorter time in the ICU before transfer as compared to the patients discharged to a ward or dead in the ICU (Table 1). Out of 134,936 patients with available mortality data (missing 3617; 2.6%), 28% of the capacity transferred patients and 21% of the non-transferred patients were deceased within 90 days after the ICU admission. The transferred patients spent longer time in intensive care and had fewer days alive outside the ICU during the 90-day period (Table 2).

**Table 1:** Baseline characteristics

Category	Variable	Capacity transfer	No transfer	*P*-value
Demographic	Age years, median (Q1;Q3)	67 (55;74)	68 (54;76)	0.004
	Women, *n* (%)	1104 (38)	55,891 (41)	< 0.001
Surgical status	No surgery, *n* (%)	2245 (77)	91,957 (68)	< 0.001
	Acute surgery, *n* (%)	462 (16)	15,973 (12)	
	Elective surgery, *n* (%)	205 (7)	27,711 (20)	
Risk scores	SAPS 3 score, mean (SD)	64 (14)	56 (16)	< 0.001
	SAPS 3 estimated mortality rate, median (Q1;Q3)	24 (11;45)	11 (3;30)	< 0.001
	SOFA on day 1, median (Q1;Q3)	8 (5;10)	6 (3;9)	< 0.001
Burden of care	NEMS, total score, median (Q1;Q3)	84 (39;180)	52 (34;88)	< 0.001
	NEMS, mean score, median (Q1;Q3)	39 (32;49)	34 (25;43)	< 0.001
Readmission	Transferred on readmission, *n* (%)	684 (23)	–	
	Any readmission during 90 days, *n* (%)	–	11,635 (9)	
Time before event	Days in the ICU, days, median (Q1;Q3)	3 (1;6)	2 (1;3)	< 0.001
Main ICU diagnosis	Infection/sepsis, except pneumonia, *n* (%)	416 (14)	13,849 (11)	< 0.001
	Malignancy, *n* (%)	6 (0)	2043 (2)	< 0.001
	Hematology, *n* (%)	5 (0)	333 (0)	0.350
	Endocrinal disease, *n* (%)	56 (2)	7628 (6)	< 0.001
	Intoxication, *n* (%)	123 (4)	9149 (7)	< 0.001
	Neurological disorder, *n* (%)	161 (6)	7303 (6)	0.666
	Cardiac disease, *n* (%)	124 (4)	7108 (6)	0.002
	Cardiac arrest, *n* (%)	309 (11)	6903 (5)	< 0.001
	Subarachnoid haemorrhage, *n* (%)	48 (2)	1302 (1)	< 0.001
	Cerebrovascular event, *n* (%)	114 (4)	3881 (3)	0.007
	Aortic rupture/dissection, *n* (%)	41 (1)	1304 (1)	0.041
	Peripheral aortic disease, *n* (%)	22 (1)	2714 (2)	< 0.001
	Musculoskeletal disorder, *n* (%)	1 (0)	92 (0)	0.451
	Shock, undefined, *n* (%)	20 (1)	2112 (2)	< 0.001
	Respiratory tract infection, incl pneumonia, *n* (%)	385 (13)	6792 (5)	< 0.001
	Airway disorder, *n* (%)	39 (1)	1328 (1)	0.116
	COPD/asthma/other respiratory disease, *n* (%)	450 (16)	11,762 (9)	< 0.001
	Acute renal failure/urological disease, *n* (%)	47 (2)	2882 (2)	0.021
	Acute abdomen, *n* (%)	80 (3)	7160 (6)	< 0.001
	Liver failure, *n* (%)	22 (1)	1066 (1)	0.643
	Pancreatitis/cholecystitis, *n* (%)	40 (1)	1393 (1)	0.147
	Psychiatric disorder, *n* (%)	1 (0)	121 (0)	0.292
	Major haemorrhage, *n* (%)	0 (0)	564 (0)	< 0.001
	Trauma, *n* (%)	181 (6)	6055 (5)	< 0.001
	Surgical complications, *n* (%)	47 (2)	3910 (3)	< 0.001
	Transplantation, *n* (%)	0 (0)	661 (1)	< 0.001
	Isolated traumatic brain injury, *n* (%)	116 (4)	3287 (3)	< 0.001
	Pregnancy related disorders, *n* (%)	0 (0)	998 (1)	< 0.001
	Postoperative care, *n* (%)	27 (1)	12,696 (10)	< 0.001
Co-morbidities	Cancer treatment, *n* (%)	140 (5)	6162 (5)	0.633
	Chronic heart failure, *n* (%)	142 (5)	7439 (6)	0.007
	Hematological malignancy, *n* (%)	64 (2)	2194 (2)	0.101
	Liver cirrhosis, *n* (%)	49 (2)	2710 (2)	0.058
	AIDS, *n* (%)	3 (0)	97 (0)	0.506
	Active cancer, *n* (%)	138 (5)	10,645 (9)	< 0.001

**Table 2:** Death rate and time alive outside the ICU within 90 days after ICU admission

Transfer due to lack of resources		No transfer	*P*-value
Dead at 30 days, *n* (%)	643 (22)	24,281 (18)	< 0.001
Dead at 90 days, *n* (%)	807 (28)	27,266 (21)	< 0.001
Days alive outside the ICU, median (Q1;Q3)	72 (11;84)	88 (81;89)	< 0.001

### Primary analysis

In the unadjusted comparison to no transfer, capacity transfer was associated with a higher risk, but in all the adjusted models, transfer was associated with a lower risk of death within 90 days after ICU admission. With adjustment for SAPS 3, ICU diagnosis and the number of days spent in the ICU before transfer or discharge to a ward/death in the ICU, and the odds ratio for death within 90 days was 0.71 (95% CI 0.65–0.79) among capacity transferred as compared to non-transferred patients (Table 3a). With adjustment for SAPS3 and ICU diagnosis, the mean number of days spent in intensive care within 90 days of the first ICU admission was 12.4 (95% CI 12.2–12.5) for transferred and 3.3 (95% CI 3.3–3.3) for non-transferred patients (Table 4a).

**Table 3:** Risk of death within 90 days after ICU admission

a All transferred patients						
Model	Cohort	OR	95% CI	*P*-value^$^	Pseudo *R*^2^	Adjusted for
Unadjusted	Transfer due to lack of resources	1.51	1.39–1.64	< 0.001	0.001	–
Model 1	Transfer due to lack of resources	0.77	0.7–0.85	< 0.001	0.371	SAPS 3
Model 2	Transfer due to lack of resources	0.71	0.64–0.78	< 0.001	0.406	SAPS 3, ICU diagnosis
Model 3	Transfer due to lack of resources	0.71	0.65–0.79	< 0.001	0.406	SAPS 3, ICU diagnosis, days in ICU
						Before transfer/discharge or death

b Inter-hospital and intra-hospital transferred patients						
Model	Cohort	OR	95% CI	*P*-value^$^	Pseudo *R*^2^	Adjusted for
Unadjusted	Inter-hospital transfer due to lack of resources	1.63	1.48–1.79	< 0.001	0.001	–
	Intra-hospital transfer due to lack of resources	1.24	1.05–1.45	0.01		
Model 1	Inter-hospital transfer due to lack of resources	0.79	0.71–0.88	< 0.001	0.371	SAPS 3
	Intra-hospital transfer due to lack of resources	0.72	0.59–0.87	< 0.001		
Model 2	Inter-hospital transfer due to lack of resources	0.75	0.67–0.84	< 0.001	0.406	SAPS 3, ICU diagnosis
	Intra-hospital transfer due to lack of resources	0.58	0.48–0.7	< 0.001		
Model 3	Inter-hospital transfer due to lack of resources	0.76	0.68–0.85	< 0.001	0.406	SAPS 3, ICU diagnosis, days in ICU
	Intra-hospital transfer due to lack of resources	0.59	0.48–0.71	< 0.001		Before transfer/discharge or death

^$^Compared to no transfer

**Table 4:** Total number of days spent in the ICU within 90 days after admission

a All transferred and non-transferred patients					
Model	Cohort	Mean	95% CI	*P*-value	Adjusted for
Unadjusted	Transfer due to lack of resources	15.3	15.1–15.4	< 0.001	–
	No transfer	3.2	3.2–3.2		–
Model 1	Transfer due to lack of resources	13.3	13.2–13.4	< 0.001	SAPS 3
	No transfer	3.3	3.3–3.3		
Model 2	Transfer due to lack of resources	12.4	12.2–12.5	< 0.001	SAPS 3, ICU
	No transfer	3.3	3.3–3.3		Diagnosis

b Inter-hospital and intra-hospital transferred and non-transferred patients					
Model	Cohort	Mean	95% CI	*P*-value	Adjusted for
Unadjusted	Inter-hospital transfer due to lack of resources	15.9	15.7–16.2	< 0.001	–
	Intra-hospital transfer due to lack of resources	15.0	14.8–15.2	< 0.001	–
	No transfer	3.2	3.2–3.2		
Model 1	Inter-hospital transfer due to lack of resources	14.2	13.9–14.5	< 0.001	SAPS 3
	Intra-hospital transfer due to lack of resources	13.0	12.8–13.1	< 0.001	
	No transfer	3.3	3.3–3.3		
Model 2	Inter-hospital transfer due to lack of resources	13.5	13.3–13.8	< 0.001	SAPS 3, ICU
	Intra-hospital transfer due to lack of resources	12.0	11.8–12.1	< 0.001	Diagnosis
	No transfer	3.3	3.3–3.3		

### Secondary analysis

In the unadjusted comparison to no transfer, both inter-hospital and intra-hospital capacity transfer were associated with a high risk, but in all the adjusted models, both inter-hospital and intra-hospital capacity transfer were associated with a lower risk of death within 90 days after ICU admission (Table 3b). With adjustment for SAPS3 and ICU diagnosis, the number of days spent in intensive care within 90 days from the first ICU admission was 13.5 (95% CI 13.3–13.8) for patients submitted to inter-hospital transfer and 12.0 (95% CI 11.8–12.1) for patients submitted to intra-hospital capacity transfer (Table 4b).

### Sensitivity and post-hoc analyses

The sensitivity and post-hoc analyses yielded no different results than the main models. The results of the sensitivity analyses which included additional adjustment of Model 3 for (1) being transferred on readmission and (2) standardised mortality rate (SMR) in transferring and receiving ICU are displayed in Additional file 1: Table S1a, b.

The result of the post-hoc analysis with controls being exposed to a lack of resources without being transferred to another ICU are displayed in Additional file 1: Table S2.

## Discussion

The main finding of this study was that unit-to-unit capacity transfer due to ICU bed shortage during 5 years before the COVID-19 pandemic in Sweden was associated with a lower risk of death within 90 days when compared to no transfer. However, capacity transferred patients had a longer total ICU stay than patients who remained for continued care in the admitting ICU.

Comparison between studies in this field is difficult due to differently categorized transfers, divergent standards of care in the referring and the receiving unit and different time points chosen for mortality analysis [14]. For the capacity transfer category as compared to no transfer, we recently found mortality not to be increased after outbound referral due to bed shortage in a Swedish university hospital ICU [11]. This agrees with a comparable study from a similar centre in Australia [15]. These results from tertiary centres are now corroborated by the present study in a wider patient population from all Swedish intensive care units. In a nation-wide study of capacity transfer in England and Wales, hospital mortality of transferred patients as compared to that of non-transferred patients was found unaffected although there was a tendency for increased level of care for transferred patients [16]. In the present study, the sensitivity and post-hoc analyses did not alter the main finding that capacity transfer was associated with a decreased risk of death 90 days after transfer due to lack of resources. Notwithstanding, a recent analysis of a Swedish national cohort, partly overlapping with our patients, demonstrated that patients exposed to capacity transfers had a higher mortality than patients transferred for other reasons [10]. The mortality after capacity transfers was similar in that cohort and ours but the comparators were different. In this study, we used an adjusted analysis for comparison to non-transferred patients with similar morbidity but remaining in the admitting ICU for further treatment. In the other study, mortality after capacity transfer was compared to the mortality after clinical and repatriation transfers. The observed higher mortality among capacity transferred patients seems reasonable in comparison to patients in the other categories. Clinical transfer to a higher level of care may be associated with better patient outcome [17]. Patients repatriated to their home ICU after specialised care may be on the way to restitution with lower sequential organ failure assessment (SOFA) score, less vasoactive drug dosage etcetera [18]. Contrarily, capacity transfers encompass patients in need of continued intensive care and expose them to a transport not for the better of their own condition. Another potential explanation for capacity transfers being associated with worse outcome than the other categories is that patients selected for transfer to another unit when bed shortage arises are sicker than patients remaining in the admitting ICU. However, in a recent report including all categories of inter-hospital transfers in Norway, the authors concluded that “patients transported between ICUs had similar morbidity and mortality rates as the rest of the ICU population” [19].

On the other hand, that capacity transfer was associated with a lower risk of death within 90 days after ICU admission when compared to no transfer, could be related to positive selection bias as the most stable patient may be selected for a non-clinical transfer when bed shortage arises [20]. In the aforementioned study of non-clinical transfers in England and Scotland, the propensity for referral was lower with increased age above 60 and a higher degree of physiological instability [16]. In addition to such a positive selection bias, being transferred out of a busy ICU may protect the patient from more risky alternatives such as a premature discharge to a ward or staying in a high workload environment, both of which are known to be associated with increased risk of death [21–23]. While a recent study found a higher mortality for transferred as compared to non-transferred patients [24], the impact of this finding on our perspective remains unclear as that study population had a low predicted mortality and as the registry providing the data did not include the reasons for transfer. Other studies that included both referred and non-referred patients did focus on clinical transfers, finding diverging results when comparing inbound referrals of a tertiary centre to direct admissions [25–27]. In the study of short-term mortality, within 24 h after inbound transfer, death was associated with specific diagnoses and not with the transport itself [18]. However, as with other examples above, no comparison was made with non-transferred patients.

In the present study, we also demonstrated that the patients transferred due to bed shortage spent more days in the ICU compared to non-transferred patients. This agrees with our earlier study in a tertiary centre as well as with other studies of patients transferred due to different reasons [11, 26]. For potential explanations, authors point at the time spent on handover, the process of assessing a new patient, different medical and/or ethical culture and lack of continuity that come with changing ICU [28, 29]. Transfer also means that medical treatment is partly brought to a pause, and the patients may need intubation for safe transportation [30, 31]. Furthermore, the receiving ICU team may assess the patient prognosis differently [32, 33].

### Strengths and limitations

The results of the present study are strengthened by our use of a national registry, covering all the Swedish ICUs, with a well-defined classification of ICU transfers, easily recognised within the Swedish organisation of health care. We report results from 5 years before the COVID-19 pandemic as we reckon that they are applicable to the current situation in spite of the surge of capacity transfers during the pandemic. No analysis was made of patients in other transfer categories as we chose to compare patients transferred due to bed shortage to patients remaining for further care in the admitting ICU. This restricts comparison to a limited number of studies in the literature but similarly designed studies support our results. The amount of missing data was low and we did not find that imputations would compensate for the small loss of information. The high quality of the data allowed us to make robust adjustments in the analysis to reach a reasonably objective comparison between transferred and non-transferred patients.

The limitations of this study include its retrospective design and that we limited the analysis to mortality and ICU length of stay. Stress factors emanating from transfer of a critically ill patient and ramming patients, families or health care professionals were not analysed but are known to affect the quality of care and potentially the outcome [34, 35]. Furthermore, the precision of the transfer category registrations was not possible to verify. The profession and experience of the persons responsible for the registry entry vary among Swedish ICUs.

Another limitation is that the external validity of our results is obscured by the varying terminology in studies and in the public debate over ICU transfers. We preferred to label the incitement of capacity transfers “bed shortage” due to the fact that capacity transfers are equalled “non-clinical transfers” in some systems, but this term may include both capacity transfers and repatriation in other countries [16]. As a matter of fact, there are numerous overlapping labels for inter-ICU transfers in the literature [20]. In our case, the term “bed shortage” in the referring ICU was chosen to illustrate our results in the public discussion over the increasing transfers due to resource limitations (https://www.theguardian.com/society/2018/mar/07/patients-turned-away-intensive-care-lack-beds-shortage-hospitals).

A final limitation of the external validity is that we could not present APACHE II data which are still used in many centres. In Sweden, SAPS3 replaced APACHE II on a national level and it is since then the only scoring system in use for the Intensive Care Registry. The SAPS3, however, includes many of the important potential confounders for outcome after intensive care including surgical status, age and co-morbidities and we did not introduce additional adjustment for these confounders into our model [36].

## Conclusion

This study showed that capacity transfer to another intensive care unit within Sweden due to a shortage of bed capacity during a 5-year period preceding the COVID-19 pandemic was associated with a lower mortality but a longer total stay in intensive care. This finding is important for the general discussion in society and for the safety assessment of ICU referrals on the international level where a shortage of intensive care capacity is common.

### Supplementary Information

**Additional file 1: Table S1.** Sensitivity analyses of the odds ratio for death at 90 days in transferred patients adding different confounders to the final model 3 described in the methods section (adjusted for SAPS 3 score, primary ICD-10 ICU diagnosis and days in the ICU before transfer/no transfer). **Table S2.** Post-hoc comparison of transferred patients limited to non-transferred patients being present in the ICU at the time of a transfer due to lack of resources (*n* = 7744). **Table S3.** Missing data. **Table S4.** Number of patients with certain diagnoses as listed in the ICD10 system.

## Data Availability

The data sets used and/or analysed during the current study are available from the corresponding author on reasonable request.

## Abbreviations

APACHE II: Acute Physiology and Chronic Health Evaluation version 2
CI: Confidence interval
COPD: Chronic obstructive pulmonary disease
Dnr: Diary number
IBM: International Business Machines Corporation
ICD-10: International Classification of Disease version 10
ICU: Intensive care unit
OR: Odds ratio
Q1;Q3: First Quartile; Third Quartile
SFAI: Swedish Society of Anaesthesia and Intensive Care
SMR: Standardised Mortality Rate
SOFA: Sequential Organ Failure Assessment Score
SAPS3: Simplified Acute Physiologic Score version 3
SD: Standard deviation
SOP: Standard operating procedure
SPSS: Statistical Package for the Social Sciences
STROBE: Strengthening the Reporting of Observational Studies in Epidemiology
vs: Versus
>: More than
