# Supplementary material for: Unit-to-unit transfer due to shortage of intensive care beds in Sweden 2015–2019 was associated with a lower risk of death but a longer intensive care stay compared to no transfer: a registry study
Source: J Intensive Care. 2024 Feb 27;12:10. doi: 10.1186/s40560-024-00722-6 (PMC10898117; doi:10.1186/s40560-024-00722-6)
Supplement: Supplementary file 1 — Additional file 1: Table S1. Sensitivity analyses of the odds ratio for death at 90 days in transferred patients adding different confounders to the final model 3 described in the methods section (adjusted for SAPS 3 score, primary ICD-10 ICU diagnosis and days in the ICU before transfer/no transfer). Table S2. Post-hoc comparison of transferred patients limited to non-transferred patients being present in the ICU at the time of a transfer due to lack of resources (n = 7744). Table S3. Missing data. Table S4. Number of patients with certain diagnoses as listed in the ICD10 system. [file 40560_2024_722_MOESM1_ESM.docx]

**Unit-to-unit transfer due to shortage of intensive care beds in Sweden 2015-2019 was associated with a lower risk of death but a longer intensive care stay compared to no transfer - a registry study**

**Additional file 1**

**Table S1**

Sensitivity analyses of the odds ratio for death at 90 days in transferred patients adding different confounders to the final model 3 described in the methods section (adjusted for SAPS 3 score, primary ICD-10 ICU diagnosis and days in the ICU before transfer/no transfer).

1. Model 3 adjusted for transfer after having been readmitted to intensive care (n=684, see table 1 in the manuscript):

| OR | 95% CI for OR | p-value | Pseudo R^2^ | Adjusted for |
| --- | --- | --- | --- | --- |
| 0.695 | 0.62 – 0.77 | <0.001 | 0.404 | SAPS 3, ICU diagnosis, days in ICU before discharge, transfer on re-admission |

1. Model 3 adjusted for Standardised Mortality Rate (SMR) in the transferring and the receiving unit. SMR is a measure of unit performance, defined by the actual mortality divided by the expected mortality according to the SAPS3 scores

| OR | 95% CI for OR | p-value | Pseudo R^2^ | Adjusted for |
| --- | --- | --- | --- | --- |
| 0.754 | 0.67 – 0.85 | <0.001 | 0.405 | SAPS 3, ICU diagnosis, days in ICU before discharge,  SMR on transferring and receiving ICU |

**Table S2**

Post-hoc comparison of transferred patients limited to non-transferred patients being present in the ICU at the time of a transfer due to lack of resources (n=7744).

| OR | 95% CI for OR | p-value | Pseudo R^2^ | Adjusted for |
| --- | --- | --- | --- | --- |
| 0.829 | 0.74 – 0.94 | 0.002 | 0.358 | SAPS 3, ICU diagnosis, days in ICU before discharge, |

**Table S3**

| **Missing data** |  |
| --- | --- |
| Variable | Missing n(%) |
| Age | 0 (0) |
| Sex | 0 (0) |
| Surgical status | 0 (0) |
| SAPS 3 score | 17486 (13) |
| SOFA score on day 1 | 118838 (86) |
| NEMS score | 104117 (75) |
| Main ICU diagnosis | 9276 (7) |

**Table S4**

**Number of patients with certain diagnoses as listed in the ICD10 system.**

| **ICD code** | **n=** | **Diagnosis** |
| --- | --- | --- |
| A020 | 2 | Infection/sepsis, except pneumonia |
| A021 | 2 | Infection/sepsis, except pneumonia |
| A039 | 1 | Infection/sepsis, except pneumonia |
| A040 | 4 | Infection/sepsis, except pneumonia |
| A042 | 3 | Infection/sepsis, except pneumonia |
| A044 | 3 | Infection/sepsis, except pneumonia |
| A047 | 34 | Infection/sepsis, except pneumonia |
| A049 | 7 | Infection/sepsis, except pneumonia |
| A072 | 1 | Infection/sepsis, except pneumonia |
| A081 | 2 | Infection/sepsis, except pneumonia |
| A090 | 7 | Infection/sepsis, except pneumonia |
| A099 | 66 | Infection/sepsis, except pneumonia |
| A150 | 2 | Infection/sepsis, except pneumonia |
| A162 | 3 | Infection/sepsis, except pneumonia |
| A170 | 1 | Infection/sepsis, except pneumonia |
| A260 | 1 | Infection/sepsis, except pneumonia |
| A267 | 2 | Infection/sepsis, except pneumonia |
| A269 | 3 | Infection/sepsis, except pneumonia |
| A280 | 1 | Infection/sepsis, except pneumonia |
| A321 | 2 | Infection/sepsis, except pneumonia |
| A329 | 1 | Infection/sepsis, except pneumonia |
| A390 | 5 | Infection/sepsis, except pneumonia |
| A392 | 4 | Infection/sepsis, except pneumonia |
| A399 | 4 | Infection/sepsis, except pneumonia |
| A400 | 45 | Infection/sepsis, except pneumonia |
| A401 | 1 | Infection/sepsis, except pneumonia |
| A402 | 3 | Infection/sepsis, except pneumonia |
| A403 | 22 | Infection/sepsis, except pneumonia |
| A408 | 14 | Infection/sepsis, except pneumonia |
| A409 | 16 | Infection/sepsis, except pneumonia |
| A410 | 68 | Infection/sepsis, except pneumonia |
| A411 | 13 | Infection/sepsis, except pneumonia |
| A412 | 4 | Infection/sepsis, except pneumonia |
| A413 | 1 | Infection/sepsis, except pneumonia |
| A414 | 6 | Infection/sepsis, except pneumonia |
| A415 | 121 | Infection/sepsis, except pneumonia |
| A418 | 22 | Infection/sepsis, except pneumonia |
| A419 | 672 | Infection/sepsis, except pneumonia |
| A449 | 2 | Infection/sepsis, except pneumonia |
| A469 | 51 | Infection/sepsis, except pneumonia |
| A480 | 7 | Infection/sepsis, except pneumonia |
| A481 | 22 | Infection/sepsis, except pneumonia |
| A483 | 4 | Infection/sepsis, except pneumonia |
| A488 | 3 | Infection/sepsis, except pneumonia |
| A490 | 5 | Infection/sepsis, except pneumonia |
| A491 | 8 | Infection/sepsis, except pneumonia |
| A498 | 7 | Infection/sepsis, except pneumonia |
| A499 | 547 | Infection/sepsis, except pneumonia |
| A709 | 2 | Infection/sepsis, except pneumonia |
| A841 | 4 | Infection/sepsis, except pneumonia |
| A848 | 1 | Infection/sepsis, except pneumonia |
| A849 | 3 | Infection/sepsis, except pneumonia |
| A858 | 2 | Infection/sepsis, except pneumonia |
| A869 | 6 | Infection/sepsis, except pneumonia |
| A878 | 1 | Infection/sepsis, except pneumonia |
| A879 | 21 | Infection/sepsis, except pneumonia |
| A985 | 2 | Infection/sepsis, except pneumonia |
| B003 | 1 | Infection/sepsis, except pneumonia |
| B004 | 36 | Infection/sepsis, except pneumonia |
| B027 | 1 | Infection/sepsis, except pneumonia |
| B088 | 1 | Infection/sepsis, except pneumonia |
| B190 | 2 | Infection/sepsis, except pneumonia |
| B199 | 1 | Infection/sepsis, except pneumonia |
| B201 | 1 | Infection/sepsis, except pneumonia |
| B232 | 1 | Infection/sepsis, except pneumonia |
| B249 | 1 | Infection/sepsis, except pneumonia |
| B250 | 2 | Infection/sepsis, except pneumonia |
| B268 | 1 | Infection/sepsis, except pneumonia |
| B279 | 2 | Infection/sepsis, except pneumonia |
| B341 | 1 | Infection/sepsis, except pneumonia |
| B349 | 27 | Infection/sepsis, except pneumonia |
| B376 | 1 | Infection/sepsis, except pneumonia |
| B377 | 8 | Infection/sepsis, except pneumonia |
| B440 | 2 | Infection/sepsis, except pneumonia |
| B441 | 1 | Infection/sepsis, except pneumonia |
| B485 | 7 | Infection/sepsis, except pneumonia |
| B499 | 2 | Infection/sepsis, except pneumonia |
| B500 | 1 | Infection/sepsis, except pneumonia |
| B508 | 5 | Infection/sepsis, except pneumonia |
| B509 | 2 | Infection/sepsis, except pneumonia |
| B549 | 13 | Infection/sepsis, except pneumonia |
| B599 | 39 | Infection/sepsis, except pneumonia |
| B670 | 1 | Infection/sepsis, except pneumonia |
| B679 | 1 | Infection/sepsis, except pneumonia |
| B950 | 8 | Infection/sepsis, except pneumonia |
| B951 | 2 | Infection/sepsis, except pneumonia |
| B952 | 1 | Infection/sepsis, except pneumonia |
| B953 | 13 | Infection/sepsis, except pneumonia |
| B955 | 2 | Infection/sepsis, except pneumonia |
| B956 | 11 | Infection/sepsis, except pneumonia |
| B957 | 3 | Infection/sepsis, except pneumonia |
| B960 | 2 | Infection/sepsis, except pneumonia |
| B961 | 2 | Infection/sepsis, except pneumonia |
| B962 | 8 | Infection/sepsis, except pneumonia |
| B963 | 3 | Infection/sepsis, except pneumonia |
| B965 | 3 | Infection/sepsis, except pneumonia |
| B968 | 36 | Infection/sepsis, except pneumonia |
| B999 | 99 | Infection/sepsis, except pneumonia |
| C009 | 1 | Malignancy |
| C019 | 4 | Malignancy |
| C022 | 1 | Malignancy |
| C028 | 1 | Malignancy |
| C029 | 2 | Malignancy |
| C030 | 3 | Malignancy |
| C031 | 5 | Malignancy |
| C039 | 3 | Malignancy |
| C040 | 1 | Malignancy |
| C048 | 1 | Malignancy |
| C049 | 3 | Malignancy |
| C060 | 2 | Malignancy |
| C068 | 2 | Malignancy |
| C069 | 17 | Malignancy |
| C079 | 2 | Malignancy |
| C080 | 1 | Malignancy |
| C099 | 1 | Malignancy |
| C102 | 1 | Malignancy |
| C109 | 1 | Malignancy |
| C131 | 1 | Malignancy |
| C139 | 6 | Malignancy |
| C148 | 2 | Malignancy |
| C153 | 1 | Malignancy |
| C154 | 4 | Malignancy |
| C155 | 10 | Malignancy |
| C158 | 2 | Malignancy |
| C159 | 35 | Malignancy |
| C160 | 3 | Malignancy |
| C162 | 1 | Malignancy |
| C168 | 1 | Malignancy |
| C169 | 3 | Malignancy |
| C171 | 2 | Malignancy |
| C180 | 4 | Malignancy |
| C181 | 4 | Malignancy |
| C182 | 3 | Malignancy |
| C183 | 1 | Malignancy |
| C184 | 5 | Malignancy |
| C186 | 1 | Malignancy |
| C187 | 12 | Malignancy |
| C188 | 1 | Malignancy |
| C189 | 10 | Malignancy |
| C199 | 2 | Malignancy |
| C209 | 8 | Malignancy |
| C220 | 5 | Malignancy |
| C221 | 5 | Malignancy |
| C227 | 1 | Malignancy |
| C229 | 6 | Malignancy |
| C239 | 2 | Malignancy |
| C240 | 2 | Malignancy |
| C241 | 2 | Malignancy |
| C248 | 1 | Malignancy |
| C249 | 1 | Malignancy |
| C250 | 10 | Malignancy |
| C251 | 2 | Malignancy |
| C252 | 1 | Malignancy |
| C254 | 1 | Malignancy |
| C257 | 1 | Malignancy |
| C258 | 3 | Malignancy |
| C259 | 22 | Malignancy |
| C260 | 5 | Malignancy |
| C268 | 1 | Malignancy |
| C269 | 4 | Malignancy |
| C320 | 1 | Malignancy |
| C321 | 1 | Malignancy |
| C329 | 2 | Malignancy |
| C339 | 1 | Malignancy |
| C348 | 1 | Malignancy |
| C349 | 14 | Malignancy |
| C379 | 1 | Malignancy |
| C383 | 1 | Malignancy |
| C384 | 1 | Malignancy |
| C390 | 1 | Malignancy |
| C399 | 1 | Malignancy |
| C411 | 2 | Malignancy |
| C412 | 4 | Malignancy |
| C438 | 1 | Malignancy |
| C439 | 2 | Malignancy |
| C442 | 1 | Malignancy |
| C443 | 2 | Malignancy |
| C449 | 1 | Malignancy |
| C480 | 5 | Malignancy |
| C482 | 1 | Malignancy |
| C491 | 1 | Malignancy |
| C495 | 1 | Malignancy |
| C499 | 1 | Malignancy |
| C509 | 1 | Malignancy |
| C549 | 1 | Malignancy |
| C559 | 2 | Malignancy |
| C569 | 14 | Malignancy |
| C579 | 2 | Malignancy |
| C619 | 4 | Malignancy |
| C629 | 1 | Malignancy |
| C649 | 3 | Malignancy |
| C659 | 1 | Malignancy |
| C678 | 1 | Malignancy |
| C679 | 1 | Malignancy |
| C700 | 1 | Malignancy |
| C710 | 1 | Malignancy |
| C711 | 1 | Malignancy |
| C712 | 4 | Malignancy |
| C716 | 2 | Malignancy |
| C719 | 5 | Malignancy |
| C720 | 1 | Malignancy |
| C729 | 41 | Malignancy |
| C739 | 4 | Malignancy |
| C740 | 1 | Malignancy |
| C749 | 1 | Malignancy |
| C751 | 1 | Malignancy |
| C760 | 1 | Malignancy |
| C762 | 5 | Malignancy |
| C763 | 1 | Malignancy |
| C770 | 4 | Malignancy |
| C774 | 1 | Hematology |
| C786 | 8 | Malignancy |
| C787 | 10 | Malignancy |
| C792 | 1 | Malignancy |
| C793 | 2 | Malignancy |
| C795 | 2 | Hematology |
| C798 | 1 | Malignancy |
| C809 | 1420 | Malignancy |
| C819 | 1 | Hematology |
| C829 | 1 | Hematology |
| C831 | 1 | Hematology |
| C838 | 1 | Hematology |
| C844 | 2 | Hematology |
| C851 | 5 | Hematology |
| C859 | 1 | Hematology |
| C880 | 1 | Hematology |
| C900 | 7 | Hematology |
| C901 | 1 | Hematology |
| C910 | 2 | Hematology |
| C911 | 3 | Hematology |
| C920 | 7 | Hematology |
| C950 | 4 | Hematology |
| C969 | 119 | Hematology |
| D001 | 1 | Malignancy |
| D010 | 1 | Malignancy |
| D101 | 1 | Malignancy |
| D107 | 1 | Malignancy |
| D134 | 1 | Malignancy |
| D136 | 2 | Malignancy |
| D141 | 1 | Malignancy |
| D165 | 1 | Malignancy |
| D320 | 8 | Malignancy |
| D330 | 2 | Malignancy |
| D332 | 2 | Malignancy |
| D333 | 3 | Malignancy |
| D334 | 1 | Malignancy |
| D339 | 36 | Malignancy |
| D350 | 2 | Malignancy |
| D352 | 1 | Malignancy |
| D357 | 1 | Malignancy |
| D361 | 1 | Malignancy |
| D369 | 8 | Malignancy |
| D370 | 4 | Malignancy |
| D372 | 1 | Malignancy |
| D374 | 2 | Malignancy |
| D376 | 2 | Malignancy |
| D379 | 1 | Malignancy |
| D380 | 3 | Malignancy |
| D381 | 1 | Malignancy |
| D383 | 1 | Malignancy |
| D385 | 1 | Malignancy |
| D391 | 3 | Malignancy |
| D414 | 1 | Malignancy |
| D420 | 3 | Malignancy |
| D429 | 1 | Malignancy |
| D430 | 9 | Malignancy |
| D431 | 2 | Malignancy |
| D434 | 1 | Malignancy |
| D439 | 38 | Malignancy |
| D440 | 1 | Malignancy |
| D444 | 1 | Malignancy |
| D464 | 1 | Malignancy |
| D469 | 3 | Malignancy |
| D472 | 1 | Malignancy |
| D487 | 2 | Malignancy |
| D489 | 44 | Malignancy |
| D500 | 1 | Hematology |
| D509 | 2 | Hematology |
| D570 | 3 | Hematology |
| D591 | 1 | Hematology |
| D593 | 5 | Hematology |
| D596 | 1 | Hematology |
| D629 | 16 | Major haemorrhage |
| D649 | 12 | Hematology |
| D659 | 1 | Hematology |
| D669 | 1 | Hematology |
| D683 | 3 | Hematology |
| D685 | 1 | Hematology |
| D689 | 52 | Hematology |
| D693 | 3 | Hematology |
| D696 | 3 | Hematology |
| D699 | 1 | Hematology |
| D709 | 20 | Hematology |
| D733 | 1 | Hematology |
| D734 | 1 | Hematology |
| D735 | 2 | Hematology |
| D738 | 3 | Hematology |
| D739 | 2 | Hematology |
| D749 | 2 | Hematology |
| D759 | 49 | Hematology |
| D761 | 3 | Hematology |
| D841 | 1 | Hematology |
| D849 | 3 | Hematology |
| D899 | 2 | Hematology |
| E035 | 18 | Endocrinal disease |
| E038 | 1 | Endocrinal disease |
| E039 | 1 | Endocrinal disease |
| E040 | 1 | Endocrinal disease |
| E049 | 1 | Endocrinal disease |
| E055 | 8 | Endocrinal disease |
| E079 | 1 | Endocrinal disease |
| E100 | 51 | Endocrinal disease |
| E101 | 349 | Endocrinal disease |
| E102 | 3 | Endocrinal disease |
| E105 | 1 | Endocrinal disease |
| E106 | 4 | Endocrinal disease |
| E108 | 88 | Endocrinal disease |
| E109 | 5 | Endocrinal disease |
| E110 | 26 | Endocrinal disease |
| E111 | 169 | Endocrinal disease |
| E112 | 1 | Endocrinal disease |
| E116 | 5 | Endocrinal disease |
| E118 | 113 | Endocrinal disease |
| E119 | 3 | Endocrinal disease |
| E131 | 10 | Endocrinal disease |
| E137 | 1 | Endocrinal disease |
| E140 | 252 | Endocrinal disease |
| E141 | 1885 | Endocrinal disease |
| E142 | 1 | Endocrinal disease |
| E149 | 3 | Endocrinal disease |
| E159 | 4 | Endocrinal disease |
| E160 | 4 | Endocrinal disease |
| E161 | 8 | Endocrinal disease |
| E162 | 18 | Endocrinal disease |
| E210 | 1 | Endocrinal disease |
| E213 | 11 | Endocrinal disease |
| E215 | 1 | Endocrinal disease |
| E222 | 52 | Endocrinal disease |
| E230 | 3 | Endocrinal disease |
| E232 | 6 | Endocrinal disease |
| E271 | 1 | Endocrinal disease |
| E272 | 7 | Endocrinal disease |
| E274 | 37 | Endocrinal disease |
| E340 | 12 | Endocrinal disease |
| E350 | 1 | Endocrinal disease |
| E358 | 2 | Endocrinal disease |
| E419 | 1 | Endocrinal disease |
| E429 | 1 | Endocrinal disease |
| E439 | 23 | Endocrinal disease |
| E512 | 5 | Endocrinal disease |
| E662 | 18 | Endocrinal disease |
| E669 | 9 | Endocrinal disease |
| E725 | 1 | Endocrinal disease |
| E802 | 1 | Endocrinal disease |
| E833 | 1 | Endocrinal disease |
| E835 | 17 | Endocrinal disease |
| E849 | 2 | Endocrinal disease |
| E858 | 3 | Endocrinal disease |
| E869 | 234 | Endocrinal disease |
| E870 | 215 | Endocrinal disease |
| E871 | 2058 | Endocrinal disease |
| E872 | 373 | Endocrinal disease |
| E873 | 59 | Endocrinal disease |
| E874 | 5 | Endocrinal disease |
| E875 | 411 | Endocrinal disease |
| E876 | 455 | Endocrinal disease |
| E877 | 15 | Endocrinal disease |
| E878 | 579 | Endocrinal disease |
| E883 | 2 | Endocrinal disease |
| E899 | 1 | Endocrinal disease |
| F001 | 1 | Neurological disorder |
| F009 | 1 | Neurological disorder |
| F050 | 5 | Neurological disorder |
| F051 | 2 | Neurological disorder |
| F058 | 36 | Neurological disorder |
| F059 | 109 | Neurological disorder |
| F061 | 1 | Neurological disorder |
| F069 | 1 | Neurological disorder |
| F099 | 1 | Neurological disorder |
| F100 | 963 | Intoxication |
| F101 | 6 | Intoxication |
| F102 | 5 | Intoxication |
| F103 | 11 | Intoxication |
| F104 | 404 | Intoxication |
| F105 | 5 | Intoxication |
| F106 | 1 | Intoxication |
| F107 | 1 | Intoxication |
| F109 | 2 | Intoxication |
| F110 | 83 | Intoxication |
| F118 | 1 | Intoxication |
| F119 | 3 | Intoxication |
| F120 | 7 | Intoxication |
| F121 | 1 | Intoxication |
| F123 | 1 | Intoxication |
| F130 | 352 | Intoxication |
| F131 | 1 | Intoxication |
| F133 | 2 | Intoxication |
| F134 | 5 | Intoxication |
| F139 | 1 | Intoxication |
| F140 | 7 | Intoxication |
| F142 | 1 | Intoxication |
| F150 | 21 | Intoxication |
| F151 | 2 | Intoxication |
| F153 | 1 | Intoxication |
| F159 | 1 | Intoxication |
| F160 | 5 | Intoxication |
| F180 | 2 | Intoxication |
| F190 | 186 | Intoxication |
| F191 | 2 | Intoxication |
| F192 | 3 | Intoxication |
| F193 | 1 | Intoxication |
| F194 | 2 | Intoxication |
| F199 | 5 | Intoxication |
| F202 | 1 | Psychiatric disorder |
| F232 | 1 | Psychiatric disorder |
| F239 | 1 | Psychiatric disorder |
| F259 | 1 | Psychiatric disorder |
| F299 | 58 | Psychiatric disorder |
| F314 | 1 | Psychiatric disorder |
| F319 | 1 | Psychiatric disorder |
| F323 | 1 | Psychiatric disorder |
| F329 | 20 | Psychiatric disorder |
| F339 | 2 | Psychiatric disorder |
| F412 | 4 | Psychiatric disorder |
| F419 | 3 | Psychiatric disorder |
| F430 | 1 | Psychiatric disorder |
| F431 | 1 | Psychiatric disorder |
| F445 | 5 | Psychiatric disorder |
| F459 | 1 | Psychiatric disorder |
| F500 | 1 | Psychiatric disorder |
| F639 | 1 | Psychiatric disorder |
| F680 | 1 | Psychiatric disorder |
| F681 | 5 | Psychiatric disorder |
| F688 | 1 | Psychiatric disorder |
| F803 | 2 | Psychiatric disorder |
| F919 | 1 | Psychiatric disorder |
| F999 | 2 | Psychiatric disorder |
| G001 | 22 | Infection/sepsis, except pneumonia |
| G002 | 4 | Infection/sepsis, except pneumonia |
| G003 | 1 | Infection/sepsis, except pneumonia |
| G008 | 19 | Infection/sepsis, except pneumonia |
| G009 | 316 | Infection/sepsis, except pneumonia |
| G021 | 1 | Infection/sepsis, except pneumonia |
| G039 | 41 | Infection/sepsis, except pneumonia |
| G040 | 1 | Infection/sepsis, except pneumonia |
| G042 | 1 | Infection/sepsis, except pneumonia |
| G048 | 1 | Infection/sepsis, except pneumonia |
| G049 | 148 | Infection/sepsis, except pneumonia |
| G051 | 2 | Infection/sepsis, except pneumonia |
| G052 | 1 | Infection/sepsis, except pneumonia |
| G060 | 26 | Infection/sepsis, except pneumonia |
| G061 | 21 | Infection/sepsis, except pneumonia |
| G062 | 4 | Infection/sepsis, except pneumonia |
| G099 | 1 | Neurological disorder |
| G109 | 1 | Neurological disorder |
| G122 | 54 | Neurological disorder |
| G209 | 5 | Neurological disorder |
| G210 | 3 | Neurological disorder |
| G239 | 1 | Neurological disorder |
| G249 | 1 | Neurological disorder |
| G319 | 1 | Neurological disorder |
| G359 | 11 | Neurological disorder |
| G378 | 1 | Neurological disorder |
| G400 | 2 | Neurological disorder |
| G401 | 4 | Neurological disorder |
| G402 | 11 | Neurological disorder |
| G403 | 9 | Neurological disorder |
| G404 | 27 | Neurological disorder |
| G406 | 10 | Neurological disorder |
| G407 | 1 | Neurological disorder |
| G408 | 3 | Neurological disorder |
| G409 | 127 | Neurological disorder |
| G410 | 32 | Neurological disorder |
| G411 | 3 | Neurological disorder |
| G412 | 18 | Neurological disorder |
| G418 | 3 | Neurological disorder |
| G419 | 808 | Neurological disorder |
| G433 | 1 | Neurological disorder |
| G440 | 1 | Neurological disorder |
| G448 | 2 | Neurological disorder |
| G451 | 1 | Neurological disorder |
| G458 | 1 | Neurological disorder |
| G459 | 5 | Neurological disorder |
| G464 | 1 | Neurological disorder |
| G468 | 1 | Neurological disorder |
| G473 | 2 | Neurological disorder |
| G479 | 2 | Neurological disorder |
| G546 | 2 | Neurological disorder |
| G549 | 1 | Neurological disorder |
| G610 | 119 | Neurological disorder |
| G629 | 3 | Neurological disorder |
| G649 | 2 | Neurological disorder |
| G700 | 72 | Neurological disorder |
| G702 | 1 | Neurological disorder |
| G709 | 1 | Neurological disorder |
| G710 | 5 | Neurological disorder |
| G713 | 1 | Neurological disorder |
| G719 | 10 | Neurological disorder |
| G723 | 1 | Neurological disorder |
| G728 | 4 | Neurological disorder |
| G732 | 1 | Neurological disorder |
| G737 | 1 | Neurological disorder |
| G819 | 1 | Neurological disorder |
| G822 | 1 | Neurological disorder |
| G825 | 3 | Neurological disorder |
| G835 | 1 | Neurological disorder |
| G838 | 7 | Neurological disorder |
| G839 | 9 | Neurological disorder |
| G904 | 1 | Neurological disorder |
| G909 | 1 | Neurological disorder |
| G911 | 4 | Neurological disorder |
| G912 | 2 | Neurological disorder |
| G918 | 2 | Neurological disorder |
| G919 | 37 | Neurological disorder |
| G929 | 1 | Neurological disorder |
| G931 | 110 | Cardiac arrest |
| G934 | 16 | Neurological disorder |
| G935 | 51 | Isolated traumatic brain injury |
| G936 | 25 | Isolated traumatic brain injury |
| G938 | 94 | Neurological disorder |
| G939 | 3 | Neurological disorder |
| G943 | 5 | Neurological disorder |
| G948 | 1 | Neurological disorder |
| G952 | 23 | Isolated traumatic brain injury |
| G959 | 7 | Isolated traumatic brain injury |
| G969 | 3 | Neurological disorder |
| G979 | 2 | Neurological disorder |
| G989 | 72 | Neurological disorder |
| G998 | 1 | Neurological disorder |
| H660 | 1 | Postoperative care |
| H812 | 1 | Postoperative care |
| H814 | 2 | Postoperative care |
| I052 | 1 | Cardiac disease |
| I059 | 1 | Cardiac disease |
| I071 | 1 | Cardiac disease |
| I109 | 110 | Cardiac disease |
| I110 | 20 | Cardiac disease |
| I120 | 4 | Cardiac disease |
| I129 | 1 | Cardiac disease |
| I130 | 1 | Cardiac disease |
| I131 | 1 | Cardiac disease |
| I132 | 8 | Cardiac disease |
| I151 | 1 | Cardiac disease |
| I158 | 7 | Cardiac disease |
| I159 | 2 | Cardiac disease |
| I200 | 5 | Cardiac disease |
| I209 | 119 | Cardiac disease |
| I210 | 10 | Cardiac disease |
| I211 | 2 | Cardiac disease |
| I212 | 6 | Cardiac disease |
| I213 | 7 | Cardiac disease |
| I214 | 13 | Cardiac disease |
| I219 | 560 | Cardiac disease |
| I229 | 4 | Cardiac disease |
| I230 | 1 | Cardiac disease |
| I238 | 2 | Cardiac disease |
| I241 | 5 | Cardiac disease |
| I248 | 2 | Cardiac disease |
| I249 | 7 | Cardiac disease |
| I250 | 1 | Cardiac disease |
| I251 | 1 | Cardiac disease |
| I255 | 4 | Cardiac disease |
| I258 | 1 | Cardiac disease |
| I259 | 11 | Cardiac disease |
| I260 | 105 | Cardiac disease |
| I269 | 748 | Cardiac disease |
| I270 | 25 | Cardiac disease |
| I272 | 2 | Cardiac disease |
| I278 | 38 | Cardiac disease |
| I279 | 3 | Cardiac disease |
| I309 | 18 | Cardiac disease |
| I312 | 5 | Cardiac disease |
| I313 | 3 | Cardiac disease |
| I319 | 57 | Cardiac disease |
| I330 | 171 | Cardiac disease |
| I339 | 3 | Cardiac disease |
| I340 | 8 | Cardiac disease |
| I350 | 141 | Cardiac disease |
| I351 | 3 | Cardiac disease |
| I359 | 1 | Cardiac disease |
| I389 | 76 | Cardiac disease |
| I398 | 1 | Cardiac disease |
| I409 | 7 | Cardiac disease |
| I420 | 10 | Cardiac disease |
| I421 | 4 | Cardiac disease |
| I422 | 1 | Cardiac disease |
| I426 | 1 | Cardiac disease |
| I428 | 2 | Cardiac disease |
| I429 | 73 | Cardiac disease |
| I441 | 3 | Cardiac disease |
| I442 | 149 | Cardiac disease |
| I443 | 1 | Cardiac disease |
| I454 | 1 | Cardiac disease |
| I455 | 2 | Cardiac disease |
| I458 | 1 | Cardiac disease |
| I459 | 27 | Cardiac disease |
| I460 | 100 | Cardiac disease |
| I469 | 6869 | Cardiac arrest |
| I471 | 3 | Cardiac disease |
| I472 | 70 | Cardiac disease |
| I479 | 2 | Cardiac disease |
| I480 | 8 | Cardiac disease |
| I481 | 4 | Cardiac disease |
| I482 | 9 | Cardiac disease |
| I483 | 1 | Cardiac disease |
| I484 | 1 | Cardiac disease |
| I489 | 303 | Cardiac disease |
| I490 | 61 | Cardiac disease |
| I495 | 18 | Cardiac disease |
| I498 | 5 | Cardiac disease |
| I499 | 132 | Cardiac disease |
| I500 | 55 | Cardiac disease |
| I501 | 902 | Cardiac disease |
| I509 | 1929 | Cardiac disease |
| I511 | 1 | Cardiac disease |
| I513 | 1 | Cardiac disease |
| I518 | 1 | Cardiac disease |
| I519 | 90 | Cardiac disease |
| I600 | 2 | Subarachnoid haemorrhage |
| I601 | 32 | Subarachnoid haemorrhage |
| I602 | 23 | Subarachnoid haemorrhage |
| I603 | 10 | Subarachnoid haemorrhage |
| I604 | 11 | Subarachnoid haemorrhage |
| I605 | 4 | Subarachnoid haemorrhage |
| I606 | 10 | Subarachnoid haemorrhage |
| I607 | 51 | Subarachnoid haemorrhage |
| I608 | 35 | Subarachnoid haemorrhage |
| I609 | 1172 | Subarachnoid haemorrhage |
| I610 | 79 | Cerebrovascular event |
| I611 | 6 | Cerebrovascular event |
| I612 | 537 | Cerebrovascular event |
| I613 | 112 | Cerebrovascular event |
| I614 | 166 | Cerebrovascular event |
| I615 | 38 | Cerebrovascular event |
| I616 | 11 | Cerebrovascular event |
| I618 | 15 | Cerebrovascular event |
| I619 | 158 | Cerebrovascular event |
| I620 | 181 | Cerebrovascular event |
| I621 | 1 | Cerebrovascular event |
| I629 | 991 | Cerebrovascular event |
| I630 | 20 | Cerebrovascular event |
| I631 | 1 | Cerebrovascular event |
| I632 | 4 | Cerebrovascular event |
| I633 | 26 | Cerebrovascular event |
| I634 | 22 | Cerebrovascular event |
| I635 | 25 | Cerebrovascular event |
| I636 | 26 | Cerebrovascular event |
| I638 | 76 | Cerebrovascular event |
| I639 | 1196 | Cerebrovascular event |
| I649 | 16 | Cerebrovascular event |
| I650 | 1 | Cerebrovascular event |
| I651 | 7 | Cerebrovascular event |
| I652 | 35 | Cerebrovascular event |
| I659 | 2 | Cerebrovascular event |
| I660 | 3 | Cerebrovascular event |
| I663 | 1 | Cerebrovascular event |
| I669 | 35 | Cerebrovascular event |
| I671 | 5 | Cerebrovascular event |
| I674 | 2 | Cerebrovascular event |
| I676 | 5 | Cerebrovascular event |
| I678 | 12 | Cerebrovascular event |
| I679 | 179 | Cerebrovascular event |
| I691 | 1 | Cerebrovascular event |
| I700 | 3 | Peripheral aortic disease |
| I702 | 25 | Peripheral aortic disease |
| I708 | 5 | Peripheral aortic disease |
| I709 | 6 | Peripheral aortic disease |
| I710 | 624 | Aortic rupture/dissection |
| I711 | 14 | Aortic rupture/dissection |
| I712 | 21 | Peripheral aortic disease |
| I713 | 109 | Aortic rupture/dissection |
| I714 | 200 | Peripheral aortic disease |
| I715 | 1 | Aortic rupture/dissection |
| I716 | 15 | Peripheral aortic disease |
| I718 | 597 | Aortic rupture/dissection |
| I719 | 1048 | Peripheral aortic disease |
| I720 | 1 | Peripheral aortic disease |
| I722 | 1 | Peripheral aortic disease |
| I723 | 12 | Peripheral aortic disease |
| I724 | 3 | Peripheral aortic disease |
| I725 | 1 | Peripheral aortic disease |
| I726 | 1 | Peripheral aortic disease |
| I728 | 4 | Peripheral aortic disease |
| I729 | 2 | Peripheral aortic disease |
| I739 | 156 | Peripheral aortic disease |
| I740 | 9 | Peripheral aortic disease |
| I742 | 6 | Peripheral aortic disease |
| I743 | 99 | Peripheral aortic disease |
| I744 | 24 | Peripheral aortic disease |
| I745 | 8 | Peripheral aortic disease |
| I748 | 18 | Peripheral aortic disease |
| I749 | 920 | Peripheral aortic disease |
| I770 | 1 | Peripheral aortic disease |
| I772 | 1 | Peripheral aortic disease |
| I778 | 1 | Peripheral aortic disease |
| I779 | 2 | Peripheral aortic disease |
| I789 | 1 | Peripheral aortic disease |
| I790 | 1 | Peripheral aortic disease |
| I792 | 2 | Peripheral aortic disease |
| I798 | 3 | Peripheral aortic disease |
| I809 | 3 | Peripheral aortic disease |
| I819 | 4 | Peripheral aortic disease |
| I822 | 3 | Peripheral aortic disease |
| I828 | 15 | Peripheral aortic disease |
| I829 | 101 | Peripheral aortic disease |
| I839 | 1 | Peripheral aortic disease |
| I850 | 231 | Liver failure |
| I859 | 3 | Liver failure |
| I864 | 2 | Peripheral aortic disease |
| I889 | 1 | Peripheral aortic disease |
| I890 | 1 | Peripheral aortic disease |
| I950 | 1 | Shock, undefined |
| I952 | 45 | Shock, undefined |
| I958 | 33 | Shock, undefined |
| I959 | 147 | Shock, undefined |
| I978 | 193 | Surgical complications |
| I979 | 4 | Surgical complications |
| I983 | 1 | Surgical complications |
| I988 | 2 | Surgical complications |
| I999 | 5 | Peripheral aortic disease |
| J020 | 1 | Shock, undefined |
| J029 | 5 | Shock, undefined |
| J030 | 4 | Shock, undefined |
| J038 | 4 | Shock, undefined |
| J039 | 17 | Respiratory tract infection, incl pneumonia |
| J040 | 9 | Shock, undefined |
| J041 | 2 | Shock, undefined |
| J042 | 121 | Respiratory tract infection, incl pneumonia |
| J050 | 7 | Shock, undefined |
| J051 | 427 | Respiratory tract infection, incl pneumonia |
| J060 | 1 | Shock, undefined |
| J068 | 5 | Shock, undefined |
| J069 | 18 | Respiratory tract infection, incl pneumonia |
| J099 | 167 | Respiratory tract infection, incl pneumonia |
| J100 | 302 | Respiratory tract infection, incl pneumonia |
| J101 | 97 | Respiratory tract infection, incl pneumonia |
| J108 | 33 | Shock, undefined |
| J121 | 73 | Respiratory tract infection, incl pneumonia |
| J122 | 1 | Shock, undefined |
| J128 | 4 | Shock, undefined |
| J129 | 90 | Respiratory tract infection, incl pneumonia |
| J139 | 83 | Respiratory tract infection, incl pneumonia |
| J149 | 18 | Shock, undefined |
| J150 | 7 | Shock, undefined |
| J151 | 3 | Shock, undefined |
| J152 | 11 | Respiratory tract infection, incl pneumonia |
| J154 | 10 | Respiratory tract infection, incl pneumonia |
| J155 | 3 | Respiratory tract infection, incl pneumonia |
| J156 | 3 | Shock, undefined |
| J157 | 13 | Shock, undefined |
| J158 | 42 | Shock, undefined |
| J159 | 3817 | Respiratory tract infection, incl pneumonia |
| J160 | 1 | Shock, undefined |
| J168 | 11 | Respiratory tract infection, incl pneumonia |
| J170 | 1 | Shock, undefined |
| J172 | 7 | Shock, undefined |
| J178 | 3 | Shock, undefined |
| J180 | 15 | Shock, undefined |
| J181 | 18 | Shock, undefined |
| J188 | 13 | Shock, undefined |
| J189 | 334 | Respiratory tract infection, incl pneumonia |
| J201 | 1 | Shock, undefined |
| J205 | 3 | Shock, undefined |
| J208 | 1 | Shock, undefined |
| J209 | 4 | Shock, undefined |
| J210 | 2 | Shock, undefined |
| J219 | 1 | Shock, undefined |
| J229 | 21 | Respiratory tract infection, incl pneumonia |
| J324 | 1 | Shock, undefined |
| J369 | 25 | Respiratory tract infection, incl pneumonia |
| J380 | 13 | Airway disorder |
| J381 | 1 | Shock, undefined |
| J384 | 496 | Airway disorder |
| J385 | 5 | Shock, undefined |
| J386 | 1 | Shock, undefined |
| J387 | 6 | Airway disorder |
| J390 | 51 | Respiratory tract infection, incl pneumonia |
| J391 | 10 | Shock, undefined |
| J393 | 4 | Shock, undefined |
| J398 | 30 | Airway disorder |
| J399 | 17 | Airway disorder |
| J409 | 1 | COPD/asthma/other respiratory disease |
| J440 | 155 | COPD/asthma/other respiratory disease |
| J441 | 430 | COPD/asthma/other respiratory disease |
| J448 | 15 | COPD/asthma/other respiratory disease |
| J449 | 2129 | COPD/asthma/other respiratory disease |
| J450 | 3 | COPD/asthma/other respiratory disease |
| J451 | 5 | COPD/asthma/other respiratory disease |
| J459 | 17 | COPD/asthma/other respiratory disease |
| J469 | 255 | COPD/asthma/other respiratory disease |
| J479 | 1 | Respiratory tract infection, incl pneumonia |
| J681 | 1 | Airway disorder |
| J682 | 1 | Airway disorder |
| J683 | 3 | Airway disorder |
| J688 | 1 | Airway disorder |
| J689 | 1 | Airway disorder |
| J690 | 1122 | Respiratory tract infection, incl pneumonia |
| J691 | 2 | Airway disorder |
| J698 | 1 | Airway disorder |
| J702 | 1 | Airway disorder |
| J704 | 2 | Airway disorder |
| J708 | 1 | Airway disorder |
| J709 | 2 | Airway disorder |
| J809 | 1257 | COPD/asthma/other respiratory disease |
| J819 | 423 | COPD/asthma/other respiratory disease |
| J841 | 190 | COPD/asthma/other respiratory disease |
| J848 | 2 | Airway disorder |
| J849 | 17 | Airway disorder |
| J851 | 3 | Respiratory tract infection, incl pneumonia |
| J853 | 1 | Respiratory tract infection, incl pneumonia |
| J869 | 79 | Respiratory tract infection, incl pneumonia |
| J909 | 17 | Respiratory tract infection, incl pneumonia |
| J919 | 2 | Airway disorder |
| J930 | 2 | Airway disorder |
| J931 | 19 | Airway disorder |
| J938 | 4 | Airway disorder |
| J939 | 309 | COPD/asthma/other respiratory disease |
| J942 | 9 | COPD/asthma/other respiratory disease |
| J948 | 67 | COPD/asthma/other respiratory disease |
| J949 | 1 | Airway disorder |
| J950 | 18 | Airway disorder |
| J951 | 277 | COPD/asthma/other respiratory disease |
| J952 | 624 | COPD/asthma/other respiratory disease |
| J953 | 1 | Airway disorder |
| J955 | 3 | Airway disorder |
| J958 | 10 | Respiratory tract infection, incl pneumonia |
| J959 | 1 | Airway disorder |
| J960 | 663 | COPD/asthma/other respiratory disease |
| J961 | 39 | COPD/asthma/other respiratory disease |
| J969 | 5278 | COPD/asthma/other respiratory disease |
| J981 | 39 | COPD/asthma/other respiratory disease |
| J982 | 1 | Airway disorder |
| J985 | 14 | COPD/asthma/other respiratory disease |
| J986 | 1 | Airway disorder |
| J988 | 2 | COPD/asthma/other respiratory disease |
| J989 | 11 | COPD/asthma/other respiratory disease |
| J990 | 1 | Airway disorder |
| K029 | 1 | Postoperative care |
| K046 | 1 | Infection/sepsis, except pneumonia |
| K047 | 2 | Infection/sepsis, except pneumonia |
| K052 | 1 | Infection/sepsis, except pneumonia |
| K088 | 1 | Postoperative care |
| K089 | 1 | Postoperative care |
| K099 | 1 | Postoperative care |
| K102 | 3 | Postoperative care |
| K112 | 2 | Postoperative care |
| K113 | 1 | Infection/sepsis, except pneumonia |
| K122 | 283 | Respiratory tract infection, incl pneumonia |
| K148 | 2 | Postoperative care |
| K149 | 1 | Postoperative care |
| K209 | 1 | Acute abdomen |
| K220 | 3 | Acute abdomen |
| K222 | 1 | Acute abdomen |
| K223 | 64 | Acute abdomen |
| K226 | 1 | Acute abdomen |
| K229 | 1 | Acute abdomen |
| K238 | 2 | Acute abdomen |
| K250 | 111 | Acute abdomen |
| K251 | 196 | Acute abdomen |
| K252 | 2 | Acute abdomen |
| K254 | 12 | Acute abdomen |
| K255 | 3 | Acute abdomen |
| K259 | 1 | Acute abdomen |
| K260 | 72 | Acute abdomen |
| K261 | 25 | Acute abdomen |
| K263 | 1 | Acute abdomen |
| K264 | 4 | Acute abdomen |
| K270 | 30 | Acute abdomen |
| K271 | 7 | Acute abdomen |
| K272 | 1 | Acute abdomen |
| K274 | 5 | Acute abdomen |
| K275 | 2 | Acute abdomen |
| K279 | 70 | Acute abdomen |
| K280 | 1 | Acute abdomen |
| K281 | 1 | Acute abdomen |
| K284 | 3 | Acute abdomen |
| K290 | 3 | Acute abdomen |
| K298 | 1 | Acute abdomen |
| K318 | 1 | Acute abdomen |
| K352 | 13 | Acute abdomen |
| K353 | 4 | Acute abdomen |
| K358 | 4 | Acute abdomen |
| K369 | 2 | Acute abdomen |
| K379 | 2 | Acute abdomen |
| K400 | 1 | Acute abdomen |
| K403 | 5 | Acute abdomen |
| K404 | 3 | Acute abdomen |
| K413 | 3 | Acute abdomen |
| K414 | 1 | Acute abdomen |
| K420 | 2 | Acute abdomen |
| K421 | 1 | Acute abdomen |
| K430 | 1 | Acute abdomen |
| K431 | 1 | Acute abdomen |
| K432 | 4 | Acute abdomen |
| K433 | 1 | Acute abdomen |
| K434 | 1 | Acute abdomen |
| K436 | 2 | Acute abdomen |
| K437 | 1 | Acute abdomen |
| K440 | 3 | Acute abdomen |
| K441 | 6 | Acute abdomen |
| K449 | 1 | Acute abdomen |
| K450 | 2 | Acute abdomen |
| K451 | 3 | Acute abdomen |
| K460 | 43 | Acute abdomen |
| K461 | 37 | Acute abdomen |
| K501 | 1 | Acute abdomen |
| K508 | 1 | Acute abdomen |
| K509 | 1 | Acute abdomen |
| K515 | 1 | Acute abdomen |
| K518 | 1 | Acute abdomen |
| K519 | 1 | Acute abdomen |
| K521 | 1 | Acute abdomen |
| K523 | 4 | Acute abdomen |
| K528 | 1 | Acute abdomen |
| K529 | 32 | Acute abdomen |
| K550 | 432 | Acute abdomen |
| K551 | 2 | Acute abdomen |
| K558 | 2 | Acute abdomen |
| K559 | 9 | Acute abdomen |
| K560 | 14 | Acute abdomen |
| K562 | 10 | Acute abdomen |
| K563 | 6 | Acute abdomen |
| K564 | 2 | Acute abdomen |
| K565 | 29 | Acute abdomen |
| K566 | 12 | Acute abdomen |
| K567 | 1075 | Acute abdomen |
| K570 | 3 | Acute abdomen |
| K572 | 27 | Acute abdomen |
| K573 | 4 | Acute abdomen |
| K574 | 1 | Acute abdomen |
| K578 | 6 | Acute abdomen |
| K579 | 1 | Acute abdomen |
| K590 | 2 | Acute abdomen |
| K592 | 1 | Acute abdomen |
| K593 | 1 | Acute abdomen |
| K610 | 4 | Acute abdomen |
| K612 | 1 | Acute abdomen |
| K613 | 1 | Acute abdomen |
| K625 | 26 | Acute abdomen |
| K630 | 9 | Acute abdomen |
| K631 | 346 | Acute abdomen |
| K632 | 3 | Acute abdomen |
| K633 | 1 | Acute abdomen |
| K638 | 2 | Acute abdomen |
| K639 | 3 | Acute abdomen |
| K650 | 415 | Acute abdomen |
| K658 | 8 | Acute abdomen |
| K659 | 20 | Acute abdomen |
| K661 | 1 | Acute abdomen |
| K668 | 1 | Acute abdomen |
| K703 | 11 | Liver failure |
| K704 | 26 | Liver failure |
| K709 | 2 | Liver failure |
| K719 | 2 | Liver failure |
| K720 | 83 | Liver failure |
| K721 | 6 | Liver failure |
| K729 | 643 | Liver failure |
| K740 | 1 | Liver failure |
| K746 | 7 | Liver failure |
| K750 | 12 | Liver failure |
| K754 | 1 | Liver failure |
| K767 | 6 | Liver failure |
| K768 | 2 | Liver failure |
| K769 | 3 | Liver failure |
| K778 | 1 | Liver failure |
| K800 | 15 | Pancreatitis/cholecystitis |
| K802 | 1 | Pancreatitis/cholecystitis |
| K803 | 25 | Pancreatitis/cholecystitis |
| K805 | 3 | Pancreatitis/cholecystitis |
| K810 | 58 | Pancreatitis/cholecystitis |
| K818 | 2 | Pancreatitis/cholecystitis |
| K819 | 158 | Pancreatitis/cholecystitis |
| K822 | 2 | Pancreatitis/cholecystitis |
| K830 | 140 | Pancreatitis/cholecystitis |
| K832 | 2 | Pancreatitis/cholecystitis |
| K838 | 2 | Pancreatitis/cholecystitis |
| K839 | 1 | Pancreatitis/cholecystitis |
| K850 | 17 | Pancreatitis/cholecystitis |
| K851 | 28 | Pancreatitis/cholecystitis |
| K852 | 27 | Pancreatitis/cholecystitis |
| K853 | 1 | Pancreatitis/cholecystitis |
| K858 | 24 | Pancreatitis/cholecystitis |
| K859 | 920 | Pancreatitis/cholecystitis |
| K861 | 2 | Pancreatitis/cholecystitis |
| K863 | 1 | Pancreatitis/cholecystitis |
| K869 | 2 | Pancreatitis/cholecystitis |
| K870 | 1 | Pancreatitis/cholecystitis |
| K871 | 1 | Pancreatitis/cholecystitis |
| K908 | 1 | Acute abdomen |
| K913 | 2 | Acute abdomen |
| K914 | 1 | Acute abdomen |
| K919 | 2 | Acute abdomen |
| K920 | 26 | Acute abdomen |
| K921 | 35 | Acute abdomen |
| K922 | 3609 | Acute abdomen |
| K928 | 1 | Acute abdomen |
| K929 | 4 | Acute abdomen |
| L009 | 1 | Infection/sepsis, except pneumonia |
| L021 | 5 | Infection/sepsis, except pneumonia |
| L023 | 1 | Infection/sepsis, except pneumonia |
| L024 | 5 | Infection/sepsis, except pneumonia |
| L029 | 3 | Infection/sepsis, except pneumonia |
| L031 | 11 | Infection/sepsis, except pneumonia |
| L032 | 2 | Infection/sepsis, except pneumonia |
| L033 | 3 | Infection/sepsis, except pneumonia |
| L038 | 1 | Infection/sepsis, except pneumonia |
| L039 | 7 | Infection/sepsis, except pneumonia |
| L088 | 4 | Infection/sepsis, except pneumonia |
| L089 | 116 | Infection/sepsis, except pneumonia |
| L232 | 1 | Infection/sepsis, except pneumonia |
| L270 | 16 | Infection/sepsis, except pneumonia |
| L401 | 1 | Infection/sepsis, except pneumonia |
| L500 | 1 | Infection/sepsis, except pneumonia |
| L512 | 2 | Infection/sepsis, except pneumonia |
| L519 | 1 | Infection/sepsis, except pneumonia |
| L892 | 1 | Infection/sepsis, except pneumonia |
| L893 | 1 | Infection/sepsis, except pneumonia |
| L899 | 6 | Infection/sepsis, except pneumonia |
| L979 | 6 | Infection/sepsis, except pneumonia |
| L989 | 5 | Infection/sepsis, except pneumonia |
| M000 | 10 | Infection/sepsis, except pneumonia |
| M002 | 6 | Infection/sepsis, except pneumonia |
| M009 | 18 | Infection/sepsis, except pneumonia |
| M051 | 1 | Infection/sepsis, except pneumonia |
| M199 | 1 | Musculoskeletal disorder |
| M311 | 2 | Musculoskeletal disorder |
| M313 | 2 | Musculoskeletal disorder |
| M317 | 1 | Musculoskeletal disorder |
| M349 | 1 | Musculoskeletal disorder |
| M354 | 1 | Musculoskeletal disorder |
| M359 | 2 | Musculoskeletal disorder |
| M402 | 1 | Musculoskeletal disorder |
| M412 | 2 | Musculoskeletal disorder |
| M414 | 3 | Musculoskeletal disorder |
| M419 | 33 | Musculoskeletal disorder |
| M459 | 1 | Musculoskeletal disorder |
| M463 | 1 | Infection/sepsis, except pneumonia |
| M464 | 2 | Infection/sepsis, except pneumonia |
| M465 | 1 | Infection/sepsis, except pneumonia |
| M468 | 1 | Musculoskeletal disorder |
| M480 | 6 | Musculoskeletal disorder |
| M485 | 1 | Musculoskeletal disorder |
| M500 | 2 | Musculoskeletal disorder |
| M501 | 2 | Musculoskeletal disorder |
| M532 | 1 | Musculoskeletal disorder |
| M538 | 1 | Musculoskeletal disorder |
| M539 | 2 | Musculoskeletal disorder |
| M600 | 3 | Infection/sepsis, except pneumonia |
| M609 | 1 | Musculoskeletal disorder |
| M622 | 1 | Musculoskeletal disorder |
| M628 | 1 | Musculoskeletal disorder |
| M632 | 1 | Musculoskeletal disorder |
| M702 | 2 | Musculoskeletal disorder |
| M726 | 167 | Infection/sepsis, except pneumonia |
| M793 | 1 | Musculoskeletal disorder |
| M796 | 9 | Musculoskeletal disorder |
| M798 | 1 | Musculoskeletal disorder |
| M840 | 1 | Musculoskeletal disorder |
| M858 | 1 | Musculoskeletal disorder |
| M860 | 1 | Infection/sepsis, except pneumonia |
| M861 | 1 | Infection/sepsis, except pneumonia |
| M869 | 9 | Infection/sepsis, except pneumonia |
| M873 | 1 | Musculoskeletal disorder |
| M878 | 4 | Musculoskeletal disorder |
| M879 | 1 | Musculoskeletal disorder |
| M907 | 1 | Musculoskeletal disorder |
| M959 | 1 | Musculoskeletal disorder |
| N009 | 1 | Acute renal failure/urological disease |
| N035 | 1 | Acute renal failure/urological disease |
| N109 | 211 | Acute renal failure/urological disease |
| N110 | 5 | Acute renal failure/urological disease |
| N111 | 4 | Acute renal failure/urological disease |
| N129 | 4 | Acute renal failure/urological disease |
| N130 | 1 | Acute renal failure/urological disease |
| N132 | 13 | Acute renal failure/urological disease |
| N136 | 29 | Acute renal failure/urological disease |
| N139 | 1 | Acute renal failure/urological disease |
| N151 | 3 | Acute renal failure/urological disease |
| N159 | 1 | Acute renal failure/urological disease |
| N170 | 15 | Acute renal failure/urological disease |
| N178 | 75 | Acute renal failure/urological disease |
| N179 | 2326 | Acute renal failure/urological disease |
| N181 | 1 | Acute renal failure/urological disease |
| N184 | 6 | Acute renal failure/urological disease |
| N185 | 19 | Acute renal failure/urological disease |
| N189 | 130 | Acute renal failure/urological disease |
| N199 | 13 | Acute renal failure/urological disease |
| N200 | 6 | Acute renal failure/urological disease |
| N201 | 6 | Acute renal failure/urological disease |
| N202 | 5 | Acute renal failure/urological disease |
| N209 | 5 | Acute renal failure/urological disease |
| N251 | 1 | Acute renal failure/urological disease |
| N288 | 1 | Acute renal failure/urological disease |
| N289 | 1 | Acute renal failure/urological disease |
| N291 | 3 | Acute renal failure/urological disease |
| N300 | 5 | Acute renal failure/urological disease |
| N309 | 2 | Acute renal failure/urological disease |
| N321 | 1 | Acute renal failure/urological disease |
| N324 | 1 | Acute renal failure/urological disease |
| N329 | 2 | Acute renal failure/urological disease |
| N390 | 677 | Infection/sepsis, except pneumonia |
| N410 | 1 | Acute renal failure/urological disease |
| N412 | 1 | Infection/sepsis, except pneumonia |
| N492 | 1 | Acute renal failure/urological disease |
| N498 | 8 | Acute renal failure/urological disease |
| N501 | 1 | Acute renal failure/urological disease |
| N619 | 1 | Postoperative care |
| N649 | 1 | Postoperative care |
| N700 | 2 | Infection/sepsis, except pneumonia |
| N709 | 1 | Infection/sepsis, except pneumonia |
| N710 | 3 | Acute abdomen |
| N768 | 1 | Postoperative care |
| N823 | 1 | Postoperative care |
| N830 | 1 | Postoperative care |
| N938 | 6 | Postoperative care |
| N939 | 52 | Major haemorrhage |
| N950 | 1 | Postoperative care |
| N981 | 2 | Postoperative care |
| N999 | 1 | Acute renal failure/urological disease |
| O001 | 1 | Pregnancy related disorders |
| O009 | 4 | Pregnancy related disorders |
| O031 | 1 | Pregnancy related disorders |
| O041 | 1 | Pregnancy related disorders |
| O081 | 1 | Pregnancy related disorders |
| O119 | 1 | Pregnancy related disorders |
| O139 | 1 | Pregnancy related disorders |
| O140 | 9 | Pregnancy related disorders |
| O141 | 230 | Pregnancy related disorders |
| O142 | 43 | Pregnancy related disorders |
| O149 | 482 | Pregnancy related disorders |
| O150 | 7 | Pregnancy related disorders |
| O151 | 3 | Pregnancy related disorders |
| O152 | 7 | Pregnancy related disorders |
| O159 | 95 | Pregnancy related disorders |
| O209 | 1 | Pregnancy related disorders |
| O230 | 3 | Pregnancy related disorders |
| O266 | 1 | Pregnancy related disorders |
| O367 | 1 | Pregnancy related disorders |
| O432 | 1 | Pregnancy related disorders |
| O441 | 1 | Pregnancy related disorders |
| O450 | 2 | Pregnancy related disorders |
| O458 | 2 | Pregnancy related disorders |
| O459 | 1 | Pregnancy related disorders |
| O460 | 1 | Pregnancy related disorders |
| O468 | 2 | Pregnancy related disorders |
| O469 | 1 | Pregnancy related disorders |
| O610 | 1 | Pregnancy related disorders |
| O670 | 2 | Pregnancy related disorders |
| O679 | 2 | Pregnancy related disorders |
| O711 | 2 | Pregnancy related disorders |
| O720 | 8 | Pregnancy related disorders |
| O721 | 32 | Pregnancy related disorders |
| O722 | 3 | Pregnancy related disorders |
| O723 | 212 | Major haemorrhage |
| O731 | 1 | Pregnancy related disorders |
| O740 | 2 | Pregnancy related disorders |
| O751 | 2 | Pregnancy related disorders |
| O753 | 1 | Pregnancy related disorders |
| O754 | 1 | Pregnancy related disorders |
| O859 | 3 | Pregnancy related disorders |
| O860 | 2 | Pregnancy related disorders |
| O861 | 1 | Pregnancy related disorders |
| O871 | 1 | Pregnancy related disorders |
| O881 | 2 | Pregnancy related disorders |
| O882 | 4 | Pregnancy related disorders |
| O903 | 1 | Pregnancy related disorders |
| O909 | 1 | Pregnancy related disorders |
| O911 | 1 | Pregnancy related disorders |
| O912 | 1 | Pregnancy related disorders |
| O994 | 18 | Pregnancy related disorders |
| O995 | 3 | Pregnancy related disorders |
| P271 | 1 | Airway disorder |
| P284 | 1 | Cardiac arrest |
| P288 | 2 | Postoperative care |
| P909 | 55 | Neurological disorder |
| P916 | 1 | Postoperative care |
| Q052 | 1 | Postoperative care |
| Q079 | 2 | Neurological disorder |
| Q185 | 1 | Postoperative care |
| Q189 | 1 | Postoperative care |
| Q203 | 1 | Postoperative care |
| Q213 | 1 | Postoperative care |
| Q249 | 2 | Postoperative care |
| Q282 | 8 | Postoperative care |
| Q349 | 1 | Postoperative care |
| Q446 | 1 | Postoperative care |
| Q459 | 1 | Postoperative care |
| Q613 | 1 | Postoperative care |
| Q623 | 3 | Acute renal failure/urological disease |
| Q649 | 1 | Postoperative care |
| Q870 | 1 | Postoperative care |
| Q899 | 1 | Postoperative care |
| R000 | 53 | Cardiac disease |
| R001 | 163 | Cardiac disease |
| R008 | 1 | Cardiac disease |
| R029 | 3 | Cardiac disease |
| R030 | 3 | Cardiac disease |
| R031 | 5 | Cardiac disease |
| R040 | 6 | Airway disorder |
| R041 | 7 | Airway disorder |
| R042 | 11 | Airway disorder |
| R048 | 4 | Airway disorder |
| R049 | 135 | Airway disorder |
| R060 | 13 | Airway disorder |
| R061 | 349 | Airway disorder |
| R068 | 4 | Airway disorder |
| R073 | 1 | Airway disorder |
| R074 | 29 | Airway disorder |
| R090 | 10 | Cardiac arrest |
| R092 | 92 | Cardiac arrest |
| R098 | 1 | Airway disorder |
| R100 | 18 | Acute abdomen |
| R103 | 1 | Acute abdomen |
| R104 | 217 | Acute abdomen |
| R119 | 1 | Acute abdomen |
| R189 | 4 | Liver failure |
| R198 | 11 | Acute abdomen |
| R201 | 1 | Postoperative care |
| R220 | 1 | Postoperative care |
| R221 | 9 | Postoperative care |
| R252 | 6 | Neurological disorder |
| R258 | 1 | Neurological disorder |
| R290 | 2 | Neurological disorder |
| R298 | 1 | Neurological disorder |
| R319 | 8 | Acute renal failure/urological disease |
| R339 | 3 | Acute renal failure/urological disease |
| R349 | 3 | Acute renal failure/urological disease |
| R398 | 1 | Acute renal failure/urological disease |
| R400 | 656 | Neurological disorder |
| R401 | 14 | Neurological disorder |
| R402 | 1491 | Neurological disorder |
| R410 | 330 | Neurological disorder |
| R464 | 1 | Neurological disorder |
| R508 | 3 | Postoperative care |
| R509 | 9 | Postoperative care |
| R519 | 4 | Neurological disorder |
| R520 | 43 | Postoperative care |
| R521 | 7 | Postoperative care |
| R522 | 9 | Neurological disorder |
| R529 | 287 | Postoperative care |
| R559 | 65 | Neurological disorder |
| R560 | 8 | Neurological disorder |
| R568 | 2904 | Neurological disorder |
| R570 | 663 | Cardiac disease |
| R571 | 723 | Shock, undefined |
| R572 | 5660 | Infection/sepsis, except pneumonia |
| R578 | 9 | Shock, undefined |
| R579 | 29 | Shock, undefined |
| R589 | 284 | Major haemorrhage |
| R600 | 7 | Postoperative care |
| R601 | 1 | Postoperative care |
| R638 | 1 | Psychiatric disorder |
| R651 | 4425 | Infection/sepsis, except pneumonia |
| R680 | 9 | Postoperative care |
| R739 | 26 | Endocrinal disease |
| R780 | 3 | Intoxication |
| R790 | 2 | Intoxication |
| R821 | 2 | Postoperative care |
| R832 | 1 | Postoperative care |
| R900 | 1 | Postoperative care |
| R919 | 1 | Postoperative care |
| R943 | 1 | Postoperative care |
| R961 | 1 | Postoperative care |
| S000 | 1 | Isolated traumatic brain injury |
| S008 | 2 | Isolated traumatic brain injury |
| S009 | 1 | Isolated traumatic brain injury |
| S010 | 1 | Isolated traumatic brain injury |
| S017 | 2 | Isolated traumatic brain injury |
| S018 | 4 | Isolated traumatic brain injury |
| S019 | 44 | Isolated traumatic brain injury |
| S020 | 1 | Isolated traumatic brain injury |
| S021 | 38 | Isolated traumatic brain injury |
| S023 | 2 | Isolated traumatic brain injury |
| S026 | 3 | Isolated traumatic brain injury |
| S027 | 6 | Isolated traumatic brain injury |
| S028 | 2 | Isolated traumatic brain injury |
| S029 | 101 | Isolated traumatic brain injury |
| S060 | 252 | Isolated traumatic brain injury |
| S061 | 7 | Isolated traumatic brain injury |
| S062 | 14 | Isolated traumatic brain injury |
| S063 | 38 | Isolated traumatic brain injury |
| S064 | 61 | Isolated traumatic brain injury |
| S065 | 523 | Isolated traumatic brain injury |
| S066 | 236 | Isolated traumatic brain injury |
| S067 | 3 | Isolated traumatic brain injury |
| S068 | 7 | Isolated traumatic brain injury |
| S069 | 1715 | Isolated traumatic brain injury |
| S070 | 1 | Isolated traumatic brain injury |
| S091 | 1 | Isolated traumatic brain injury |
| S097 | 2 | Isolated traumatic brain injury |
| S099 | 3 | Isolated traumatic brain injury |
| S100 | 3 | Isolated traumatic brain injury |
| S101 | 1 | Isolated traumatic brain injury |
| S109 | 1 | Isolated traumatic brain injury |
| S110 | 3 | Isolated traumatic brain injury |
| S117 | 2 | Isolated traumatic brain injury |
| S118 | 3 | Isolated traumatic brain injury |
| S119 | 4 | Isolated traumatic brain injury |
| S120 | 4 | Isolated traumatic brain injury |
| S121 | 15 | Isolated traumatic brain injury |
| S122 | 16 | Isolated traumatic brain injury |
| S127 | 4 | Isolated traumatic brain injury |
| S129 | 178 | Isolated traumatic brain injury |
| S131 | 1 | Isolated traumatic brain injury |
| S134 | 1 | Isolated traumatic brain injury |
| S140 | 1 | Isolated traumatic brain injury |
| S141 | 44 | Isolated traumatic brain injury |
| S150 | 1 | Isolated traumatic brain injury |
| S152 | 2 | Isolated traumatic brain injury |
| S157 | 3 | Isolated traumatic brain injury |
| S159 | 36 | Isolated traumatic brain injury |
| S170 | 4 | Isolated traumatic brain injury |
| S179 | 1 | Isolated traumatic brain injury |
| S189 | 1 | Isolated traumatic brain injury |
| S198 | 2 | Isolated traumatic brain injury |
| S199 | 1 | Isolated traumatic brain injury |
| S202 | 3 | Trauma |
| S211 | 2 | Trauma |
| S212 | 2 | Trauma |
| S217 | 1 | Trauma |
| S218 | 1 | Trauma |
| S220 | 46 | Trauma |
| S221 | 5 | Trauma |
| S222 | 80 | Trauma |
| S223 | 11 | Trauma |
| S224 | 202 | Trauma |
| S225 | 1 | Trauma |
| S241 | 5 | Trauma |
| S250 | 2 | Trauma |
| S252 | 1 | Trauma |
| S258 | 1 | Trauma |
| S259 | 13 | Trauma |
| S260 | 5 | Trauma |
| S269 | 7 | Trauma |
| S270 | 349 | Trauma |
| S271 | 156 | Trauma |
| S272 | 16 | Trauma |
| S273 | 38 | Trauma |
| S278 | 1 | Trauma |
| S279 | 1 | Trauma |
| S297 | 1 | Trauma |
| S298 | 2 | Trauma |
| S301 | 2 | Trauma |
| S307 | 1 | Trauma |
| S310 | 1 | Trauma |
| S311 | 3 | Trauma |
| S312 | 1 | Trauma |
| S318 | 1 | Trauma |
| S320 | 5 | Trauma |
| S323 | 4 | Trauma |
| S324 | 5 | Trauma |
| S325 | 1 | Trauma |
| S327 | 8 | Trauma |
| S328 | 134 | Trauma |
| S341 | 3 | Trauma |
| S352 | 1 | Trauma |
| S357 | 1 | Trauma |
| S359 | 36 | Trauma |
| S360 | 77 | Trauma |
| S361 | 21 | Trauma |
| S362 | 1 | Trauma |
| S363 | 3 | Trauma |
| S364 | 10 | Trauma |
| S365 | 3 | Trauma |
| S366 | 1 | Trauma |
| S367 | 3 | Trauma |
| S368 | 1 | Trauma |
| S369 | 311 | Trauma |
| S370 | 11 | Trauma |
| S372 | 1 | Trauma |
| S379 | 21 | Trauma |
| S390 | 1 | Trauma |
| S396 | 1 | Trauma |
| S420 | 1 | Trauma |
| S422 | 3 | Trauma |
| S451 | 1 | Trauma |
| S481 | 1 | Trauma |
| S520 | 1 | Trauma |
| S619 | 1 | Trauma |
| S708 | 1 | Trauma |
| S711 | 5 | Trauma |
| S720 | 60 | Trauma |
| S721 | 5 | Trauma |
| S722 | 2 | Trauma |
| S723 | 8 | Trauma |
| S724 | 2 | Trauma |
| S727 | 2 | Trauma |
| S728 | 1 | Trauma |
| S729 | 3 | Trauma |
| S730 | 1 | Trauma |
| S759 | 1 | Trauma |
| S821 | 2 | Trauma |
| S823 | 1 | Trauma |
| S824 | 1 | Trauma |
| S827 | 2 | Trauma |
| S828 | 2 | Trauma |
| S851 | 1 | Trauma |
| S881 | 2 | Trauma |
| S889 | 1 | Trauma |
| S899 | 1 | Trauma |
| S920 | 1 | Trauma |
| S929 | 1 | Trauma |
| S998 | 1 | Trauma |
| T021 | 1 | Trauma |
| T029 | 3 | Trauma |
| T049 | 1 | Trauma |
| T061 | 1 | Trauma |
| T068 | 2 | Trauma |
| T079 | 3521 | Trauma |
| T093 | 1 | Trauma |
| T109 | 31 | Trauma |
| T119 | 28 | Trauma |
| T129 | 122 | Trauma |
| T139 | 43 | Trauma |
| T140 | 7 | Trauma |
| T141 | 12 | Trauma |
| T149 | 200 | Trauma |
| T172 | 2 | Airway disorder |
| T173 | 1 | Airway disorder |
| T174 | 1 | Airway disorder |
| T175 | 5 | Airway disorder |
| T179 | 64 | Airway disorder |
| T181 | 12 | Acute abdomen |
| T189 | 4 | Airway disorder |
| T200 | 6 | Trauma |
| T201 | 1 | Trauma |
| T202 | 7 | Trauma |
| T210 | 1 | Trauma |
| T212 | 3 | Trauma |
| T213 | 3 | Trauma |
| T230 | 1 | Trauma |
| T233 | 1 | Trauma |
| T243 | 1 | Trauma |
| T270 | 1 | Trauma |
| T271 | 3 | Trauma |
| T273 | 59 | Airway disorder |
| T277 | 8 | Trauma |
| T286 | 3 | Trauma |
| T290 | 2 | Trauma |
| T293 | 3 | Trauma |
| T300 | 158 | Trauma |
| T302 | 1 | Trauma |
| T303 | 6 | Trauma |
| T304 | 5 | Trauma |
| T311 | 1 | Trauma |
| T312 | 2 | Trauma |
| T313 | 5 | Trauma |
| T314 | 1 | Trauma |
| T316 | 1 | Trauma |
| T318 | 1 | Trauma |
| T357 | 3 | Trauma |
| T365 | 1 | Intoxication |
| T381 | 2 | Intoxication |
| T383 | 23 | Intoxication |
| T388 | 1 | Intoxication |
| T390 | 18 | Intoxication |
| T391 | 327 | Intoxication |
| T393 | 18 | Intoxication |
| T398 | 8 | Intoxication |
| T399 | 8 | Intoxication |
| T400 | 4 | Intoxication |
| T401 | 71 | Intoxication |
| T402 | 611 | Intoxication |
| T403 | 5 | Intoxication |
| T404 | 5 | Intoxication |
| T405 | 1 | Intoxication |
| T406 | 328 | Intoxication |
| T407 | 26 | Intoxication |
| T408 | 2 | Intoxication |
| T409 | 11 | Intoxication |
| T413 | 2 | Intoxication |
| T414 | 17 | Intoxication |
| T421 | 1 | Intoxication |
| T423 | 5 | Intoxication |
| T424 | 1023 | Intoxication |
| T425 | 1 | Intoxication |
| T426 | 22 | Intoxication |
| T427 | 774 | Intoxication |
| T430 | 168 | Intoxication |
| T431 | 1 | Intoxication |
| T432 | 509 | Intoxication |
| T433 | 23 | Intoxication |
| T434 | 5 | Intoxication |
| T435 | 288 | Intoxication |
| T436 | 155 | Intoxication |
| T438 | 9 | Intoxication |
| T439 | 214 | Intoxication |
| T440 | 4 | Intoxication |
| T445 | 2 | Intoxication |
| T447 | 69 | Intoxication |
| T449 | 5 | Intoxication |
| T451 | 1 | Intoxication |
| T454 | 4 | Intoxication |
| T455 | 7 | Intoxication |
| T459 | 2 | Intoxication |
| T460 | 18 | Intoxication |
| T461 | 29 | Intoxication |
| T462 | 4 | Intoxication |
| T464 | 2 | Intoxication |
| T465 | 5 | Intoxication |
| T507 | 2 | Intoxication |
| T509 | 102 | Intoxication |
| T510 | 38 | Intoxication |
| T511 | 7 | Intoxication |
| T512 | 27 | Intoxication |
| T518 | 1 | Intoxication |
| T519 | 14 | Intoxication |
| T523 | 45 | Intoxication |
| T529 | 4 | Intoxication |
| T543 | 2 | Intoxication |
| T549 | 15 | Intoxication |
| T559 | 2 | Intoxication |
| T573 | 2 | Intoxication |
| T589 | 83 | Intoxication |
| T596 | 1 | Intoxication |
| T597 | 1 | Intoxication |
| T598 | 1 | Intoxication |
| T599 | 61 | Intoxication |
| T620 | 2 | Intoxication |
| T629 | 1 | Intoxication |
| T630 | 98 | Intoxication |
| T634 | 16 | Intoxication |
| T650 | 2 | Intoxication |
| T658 | 6 | Intoxication |
| T659 | 828 | Intoxication |
| T669 | 1 | Trauma |
| T670 | 10 | Postoperative care |
| T689 | 382 | Postoperative care |
| T702 | 2 | Trauma |
| T703 | 4 | Trauma |
| T719 | 65 | Cardiac arrest |
| T733 | 1 | Trauma |
| T741 | 1 | Trauma |
| T749 | 9 | Trauma |
| T751 | 65 | Cardiac arrest |
| T780 | 4 | Shock, undefined |
| T782 | 432 | Shock, undefined |
| T783 | 67 | Shock, undefined |
| T784 | 171 | Shock, undefined |
| T789 | 1 | Shock, undefined |
| T791 | 1 | Trauma |
| T792 | 90 | Trauma |
| T793 | 9 | Trauma |
| T794 | 1 | Trauma |
| T796 | 132 | Trauma |
| T797 | 21 | Trauma |
| T798 | 2 | Trauma |
| T799 | 2 | Trauma |
| T800 | 1 | Surgical complications |
| T802 | 1 | Surgical complications |
| T803 | 2 | Surgical complications |
| T808 | 3 | Surgical complications |
| T809 | 23 | Surgical complications |
| T810 | 1455 | Surgical complications |
| T811 | 216 | Shock, undefined |
| T812 | 147 | Surgical complications |
| T813 | 26 | Surgical complications |
| T814 | 234 | Infection/sepsis, except pneumonia |
| T817 | 1 | Surgical complications |
| T818 | 51 | Surgical complications |
| T819 | 207 | Surgical complications |
| T823 | 3 | Surgical complications |
| T827 | 3 | Surgical complications |
| T828 | 2 | Surgical complications |
| T845 | 3 | Surgical complications |
| T847 | 1 | Surgical complications |
| T850 | 18 | Surgical complications |
| T857 | 1 | Surgical complications |
| T861 | 2 | Surgical complications |
| T863 | 1 | Surgical complications |
| T881 | 1 | Surgical complications |
| T883 | 7 | Surgical complications |
| T884 | 100 | Surgical complications |
| T885 | 76 | Surgical complications |
| T886 | 14 | Surgical complications |
| T887 | 197 | Surgical complications |
| T888 | 5 | Surgical complications |
| T889 | 1340 | Surgical complications |
| T910 | 1 | Postoperative care |
| T969 | 6 | Postoperative care |
| U822 | 7 | Infection/sepsis, except pneumonia |
| U839 | 1 | Infection/sepsis, except pneumonia |
| Y409 | 18 | Postoperative care |
| Y423 | 43 | Intoxication |
| Y433 | 11 | Intoxication |
| Y442 | 22 | Postoperative care |
| Y445 | 10 | Intoxication |
| Y450 | 128 | Intoxication |
| Y479 | 104 | Intoxication |
| Y575 | 20 | Intoxication |
| Y639 | 16 | Surgical complications |
| Y699 | 49 | Surgical complications |
| Y832 | 79 | Postoperative care |
| Z030 | 2 | Infection/sepsis, except pneumonia |
| Z032 | 3 | Psychiatric disorder |
| Z033 | 6 | Neurological disorder |
| Z034 | 34 | Cardiac disease |
| Z035 | 2 | Cardiac disease |
| Z036 | 656 | Intoxication |
| Z038 | 32 | Postoperative care |
| Z039 | 25 | Postoperative care |
| Z041 | 8 | Trauma |
| Z042 | 1 | Trauma |
| Z043 | 6 | Trauma |
| Z045 | 3 | Trauma |
| Z048 | 21 | Postoperative care |
| Z049 | 11511 | Postoperative care |
| Z090 | 1 | Postoperative care |
| Z098 | 1 | Postoperative care |
| Z099 | 1 | Postoperative care |
| Z321 | 1 | Postoperative care |
| Z358 | 1 | Postoperative care |
| Z421 | 1 | Postoperative care |
| Z488 | 7 | Postoperative care |
| Z489 | 122 | Postoperative care |
| Z511 | 1 | Malignancy |
| Z518 | 3 | Postoperative care |
| Z711 | 1 | Postoperative care |
| Z721 | 1 | Intoxication |
| Z743 | 33 | Postoperative care |
| Z811 | 1 | Postoperative care |
| Z813 | 1 | Postoperative care |
| Z851 | 1 | Malignancy |
| Z859 | 3 | Malignancy |
| Z866 | 1 | Neurological disorder |
| Z884 | 1 | Surgical complications |
| Z892 | 1 | Trauma |
| Z903 | 1 | Postoperative care |
| Z914 | 2 | Psychiatric disorder |
| Z930 | 1 | Airway disorder |
| Z933 | 1 | Postoperative care |
| Z941 | 15 | Transplantation |
| Z942 | 36 | Transplantation |
| Z944 | 44 | Liver failure |
| Z948 | 3 | Transplantation |
| Z949 | 531 | Transplantation |
| Z988 | 76 | Transplantation |
| Z991 | 1 | Airway disorder |
| Z992 | 1 | Acute renal failure/urological disease |
